# Development of a QSAR model for predicting PPARα activation by PFAS based on human in vitro data of a comprehensive panel of legacy and novel PFAS

**DOI:** 10.1007/s00204-026-04359-2

**Published:** 2026-03-31

**Authors:** Wiebke Alker, Periklis Tsiros, Haralambos Sarimveis, Albert Braeuning, Thorsten Buhrke

**Affiliations:** 1 Department Chemical and Product Safety, German Federal Institute for Risk Assessment (BfR), Max-Dohrn-Str. 8-10, 10589 Berlin, Germany; 2 School of Chemical Engineering, National Technical University of Athens, 9 Iroon Polytechniou Str, 15772 Athens, Greece

**Keywords:** PFAS, QSAR, PPARα, BMD

## Abstract

Per- and polyfluoroalkyl substances (PFAS) are a large family of persistent environmental contaminants. Some PFAS are known to bioaccumulate, are frequently detected in human serum, and are associated with several adverse effects on the immune system, the endocrine system, and the liver. PFAS-mediated activation of the peroxisome proliferator-activated receptor alpha (PPARα), which plays a key role in lipid and cholesterol homeostasis, is suggested to be an important molecular initiating event triggering PFAS toxicity. The aim of this study was to evaluate the PPARα activation potential of a diverse panel of 34 PFAS congeners, consisting of both legacy and novel compounds, using a PPARα-dependent transactivation assay in transfected HEK293T cells. The resulting concentration-response data were analyzed using benchmark dose (BMD) modelling to quantify PPARα activation potency. A key finding was that PFAS with a sulfonic acid group showed a lower potency compared to those with a carboxylic group. The most potent activators belonged to the perfluoroalkylether carboxylic acid (PFECA) subgroup. Computational descriptors were generated to characterize each congener, and quantitative structure-activity relationship (QSAR) modelling was applied to relate molecular features to in vitro PPARα activation potency, as expressed by BMD estimates. For prioritization purposes in the context of PFAS hazard characterization, the QSAR model was used to screen about 10,000 PFAS congeners. Of these, roughly 10% were within the defined applicability domain of the developed QSAR model. Predicted BMD concentrations for PPARα activation were in the range between 0.3 µM and 420 µM.

**Supplementary Information:**

The online version contains supplementary material available at 10.1007/s00204-026-04359-2.

## Introduction

Per- and polyfluoroalkyl substances (PFAS) are synthetic fluorinated compounds known for their exceptional chemical and thermal stability. A chemical database by the U.S Environmental Protection Agency lists more than 10,000 PFAS, with a continuously increasing number (Williams et al. 2022). Due to their properties, PFAS are used in almost all industrial branches and many consumer applications, including textile coatings, food packaging, and fire-fighting foams (Glüge et al. 2020). However, the stability of the carbon-fluorine bond also contributes to their persistence in the environment, thereby causing a constant exposure of wildlife and humans with PFAS (Bangma et al. 2022; Cousins et al. 2020). PFAS accumulate in the body, mainly in the liver and the serum, as has been shown for different species and confirmed in humans by analysis of autopsy tissues (ATSDR 2021; Olsen et al. 2003; Pérez et al. 2013). According to the European Food Safety Authority (EFSA) Panel on Contaminants in the Food Chain, epidemiological studies provide evidence for an association between exposure to certain PFAS and effects on the immune system, increased serum cholesterol levels, increased serum levels of alanine transferase and reduced birth weight (EFSA 2020). The Agency for Toxic Substances and Disease Registry (ATSDR) lists the same outcomes in humans, as well as pregnancy-induced hypertension/ pre-eclampsia (ATSDR 2021). However, both reports point out that causality of the described effects still remains unclear. The International Agency for Research on Cancer (IARC) classified perfluorooctanoic acid (PFOA) as “carcinogenic to humans” (group 1) and perfluorooctanesulfonic acid (PFOS) as “possibly carcinogenic to humans” (group 2B) (IARC 2025). Further health outcomes discussed in the context of PFAS exposure include liver damage, thyroid dysfunction, reduced kidney function, and developmental toxicity (Fenton et al. 2021).

In response to growing knowledge about the persistence, bioaccumulation and adverse health effects, the use of many so called legacy PFAS (e.g., PFOA, PFOS) has been reduced, due to legal restrictions as well as voluntary actions by manufacturers (ECHA 2025; Scheringer et al. 2014). As a replacement, so called novel or alternative PFAS are increasingly used, e.g., short-chain PFAS or PFAS with a modified carbon backbone such as (poly)-ether PFAS. They are designed to retain the functional benefits of their predecessors while aiming to have reduced persistence, accumulation potential and fewer adverse health effects. Several studies have detected novel PFAS in environmental, wildlife and human samples (Brase et al. 2021; Guillette et al. 2020, 2022; Kotlarz et al. 2020; Mahoney et al. 2022; Munoz et al. 2019; Poothong et al. 2017; Scheringer et al. 2014). The actual exposure can be expected to be even higher than what has been reported, because not many alternative PFAS are routinely analyzed (e.g., due to the lack of standards and validated analytical methods) (Munoz et al. 2019). At the same time, little or no data is available on their toxicity. Several studies on alternative PFAS report effects in vivo in different species, e.g., in mice, fish and algae. Some of them compare the observed effects with those of legacy PFAS. The results differ depending on the substance and the endpoint with the effects of the tested novel PFAS being less, comparable or more severe than those of the legacy PFAS (Mahoney et al. 2022; Munoz et al. 2019; Rice et al. 2021; Sheng et al. 2018a, b; Zhang et al. 2021). Taken together, this shows the urgent need to deepen our understanding on possible modes of action of novel PFAS. The large number of PFAS makes it impossible to perform hazard characterization on all of them based on experimental data. Leveraging in silico models can offer valuable insights and guide prioritization on subsequent experiments by establishing relationships between specific structural features and observed effects.

The peroxisome proliferator-activated receptor alpha (PPARα) is a nuclear receptor with an essential role in lipid metabolism, lipoprotein metabolism, gluconeogenesis and bile acid metabolism. It is activated by endogenous molecules, e.g., natural fatty acids and eicosanoids, but also synthetic compounds like hypolipidemic drugs (Krey et al. 1997). PPARα is expressed in several tissues including the liver, kidney, heart and intestine (Christofides et al. 2021; Kersten and Stienstra 2017).

Numerous in vitro studies have shown that many PFAS activate human, rat and mouse PPARα. These studies indicate that structural characteristics (e.g., functional group, carbon chain length) seem to affect the potency of PFAS for PPARα activation (Barutcu et al. 2024; Behr et al. 2020; Bjork and Wallace 2009; Buhrke et al. 2015; Evans et al. 2022; Houck et al. 2021; Rosenmai et al. 2018; Sadrabadi et al. 2023). In vivo studies with rats show effects of PFAS exposure on PPARα related endpoints, e.g., increased mRNA levels of PPARα-dependent target genes or increased levels or activity of certain enzymes (Kudo et al. 2000; NTP 2019a, b). Overall, these findings support the notion that PPARα represents a biological target affected by exposure to certain PFAS. Its PFAS-mediated activation is assumed to be associated with the observed PFAS-induced disturbances in lipid homeostasis.

In the present study, a diverse set of 34 PFAS congeners was selected to investigate their impact on PPARα activity. The panel represents five major subgroups, including both legacy and novel PFAS: perfluoroalkyl carboxylic acids (PFCA), perfluoroalkylether carboxylic acids (PFECA), perfluoroalkylether sulfonic acids (PFESA), perfluoroalkyl sulfonic acids (PFSA), and fluorotelomer sulfonic acids (FTS). In vitro dose-response data were generated using a PPARα-dependent reporter gene assay, and Benchmark Dose (BMD) modelling was applied to those data. The resulting benchmark response (BMR) values were then analysed using quantitative structure-activity relationship (QSAR) modelling to identify patterns linking structural features of the compounds to their observed in vitro effects on PPARα activation. The QSAR model was further used to predict BMR values for PPARα activation for more than 10,000 PFAS congeners, of which approximately 10% fell within the applicability domain (AD) of the model. Assuming that PPARα activation is a relevant driver for PFAS toxicity, the BMR predictions provide a basis for prioritizing PFAS with a high activation potential for subsequent experimental toxicity testing.

## Materials and methods

### Chemicals

The 34 PFAS congeners were purchased at the highest available purity. 11-Chloroeicosafluoro-3-oxaundecane-1-sulfonic acid potassium salt (8:2 Cl-PFESA) was purchased from eNovation Chemicals (Green Brook, USA). 2,3,3,3-Tetrafluoro-2-[1,1,2,3,3,3-hexafluoro-2-(trifluoromethoxy)propoxy]propanoic acid (Branched ADONA), methyl perfluoro-3,6,9-trioxatridecanoate (CH3-PFO3TriDA), perfluoroheptanesulfonic acid (PFHpS), perfluoro-3,6-dioxadecanoic acid (PFO2DA), perfluoro-3,6-dioxaheptanoic acid (PFO2HpA), perfluoro-3,6,9-trioxadecanoic acid (PFO3DA), perfluoro-3,6,9-trioxatridecanoic acid (PFO3TriDA ), ammonium 2-perfluoropentoxy-2,3,3,3-tetrafluoropropanoate (PFoxaOA), perfluoropentanesulfonic acid (PFPeS) and potassium perfluoro(4-methyl-3,6-dioxaoctane)sulfonate (similar to Nafion-BP2) were purchased from Apollo Scientific (Manchester, UK). 2,3,3,3-Tetrafluoro-2-(heptafluoropropoxy)propanoic acid (HFPO-DA), 2,3,3,3-tetrafluoro-2-(1,1,2,3,3,3-hexafluoro-2-(perfluoropropoxy)propoxy)propanoic acid (HFPO-TA), 3,3,4,4,5,5,6,6,7,7,8,8,8-tridecafluorooctanesulfonic acid (6:2 FTS), ammonium perfluoro-3,6-dioxaoctanoate (EEA), perfluoro-2,5,8-trimethyl-3,6,9-trioxadodecanoic acid (HFPO-TeA), perfluoro-3,6-dioxa-4-methyl-7-octene-1-sulfonic acid (Nafion-BP1), 7H-perfluoro-4-methyl-3,6-dioxaoctanesulfonic acid (Nafion-BP2), perfluoro-(3-oxapentane-1-sulfonic acid) (PF2EOESA), perfluoro-3-methoxypropanoic acid (PFMOPrA) and perfluoro-4-methoxybutanoic acid (PFMOBA) were purchased from SynQuest Laboratories (Alachua, USA). Perfluorobutanoic acid (PFBA), perfluorobutanesulfonic acid (PFBS), perfluorodecanoic acid (PFDA), perfluoroheptanoic acid (PFHpA), perfluorohexanoic acid (PFHxA), perfluorohexanesulfonic acid potassium salt (PFHxS), perfluorononanoic acid (PFNA), perfluorooctanoic acid (PFOA), perfluorooctanesulfonic acid potassium salt (PFOS), perfluoropentanoic acid (PFPeA), pentafluoropropionic acid (PFPrA) and perfluoroundecanoic acid (PFUnA) were purchased from Sigma-Aldrich (Taufkirchen, Germany). 2,2-Difluoro-2-(trifluoromethoxy) acetic acid (PFMOAA) was purchased from Enamine (Frankfurt am Main, Germany). The structures of these PFAS congeners and the CAS numbers can be found in Supplementary Table 1.

### Cell culture conditions

HEK293T cells (European Collection of Cell Cultures, Porton Down, UK) were cultivated in Dulbeccos’s modified Eagles’s medium with phenol red (DMEM) (P04-03590, PAN-Biotech, Aidenbach, Germany), supplemented with 10% (v/v) fetal bovine serum (FBS) (FBS Superior, Batch #0001637726, Sigma Aldrich, Taufkirchen, Germany), 100 U/mL Penicillin and 100 µg/mL Streptomycin (Capricorn Scientific, Ebsdorfergrund, Germany) at 37 °C in a humidified atmosphere with 5% CO_2_. Cells were passaged at 80–90% confluence.

### Cytotoxicity assessment of PFAS

Cellular viability of HEK293T cells was determined by means of the 3-(4,5-dimethylthiazol-2-yl)-2,5-diphenyltetrazolium bromide (MTT) assay. 24 h prior to incubation, HEK293T cells were seeded into a 96-well-plate (20.000 cells/well). Cells were incubated with different PFAS concentrations for 24 h at 37 °C in a humidified atmosphere with 5% CO_2_. Subsequently MTT-solution (final concentration per well 0.05 mg/mL) was added to the wells, cells were incubated for 1 h at 37 °C in a humidified atmosphere with 5% CO_2_, plates were centrifuged at 300 x g for 5 min, incubation solution was aspirated and 130 µL desorption-solution (0.7% sodium dodecyle sulfate in propan-2-ol) per well were added. After 15 min shaking in the dark (microplate shaker, 600 U/min), absorption was measured at 570 nm (reference wavelength 630 nm), using a microplate reader (Infinite M Flex, Tecan). Three independent biological replicates with three technical replicates were performed. 0.01% Triton-X 100 served as positive control (PC). Cellular viability of treated cells was calculated relative to the untreated negative (solvent) control (SC).

### Dual luciferase reporter gene assay

The principle of the PPARα-dependent transactivation assay as well as the design of the used expression plasmid for PPARα (pGAL4-PPARα-LBD), the firefly luciferase (FLuc) reporter plasmid (pGAL4-(UAS)5-TK-Luc) and the *Renilla* luciferase (RLuc) plasmid (pcDNA3-Rluc) as internal control for normalization have been described before by Luckert et al. (2013, 2018). The assay relies on the proteins that are expressed from the transfected plasmids and is independent of the cell line. Therefore, HEK293T cells were used for this assay, because they are easy to handle and to transfect.

HEK293T cells (18.000 cells/well in 100 µL supplemented medium) were seeded into a 96-well-plate. After 16 h at 37 °C in a humidified atmosphere with 5% CO_2_, cells were transiently co-transfected by adding 10 µL transfection solution per well, containing 40 ng pGAL4-PPARα-LBD, 40 ng pGAL4-(UAS)5-TK-Luc, 1 ng pcDNA3-Rluc, 0.243 µL TransIT-LT (Mirus Bio, Madison, USA) in Opti-MEM (Gibco, New York, USA). After 24 h at 37 °C in a humidified atmosphere with 5% CO_2_, the medium was aspirated and cells were incubated with different PFAS concentrations in supplemented medium for 24 h at 37 °C in a humidified atmosphere with 5% CO_2_. Subsequently, incubation solution was aspirated, 50 µL/well lysis buffer (100 mM KH_2_PO_4_, 0.2% Triton-X 100 in MilliQ water, pH 7.8) were added, plates were shaken for 15 min in the dark (microplate shaker, 600 U/min) and then stored at -80 °C until measurement of FLuc and RLuc luminescence.

To measure the FLuc and RLuc luminescence, plates were put in a dark place at room temperature until the lysis buffer in the wells has thawed, shaken for 15 min in the dark (microplate shaker, 600 U/min) and then centrifuged at 300 x g for 5 min. Of each well, 5 µL lysis buffer were transferred into a white 96-well plate. The subsequent measurement of the FLuc and RLuc luminescence was done as described before by Hampf and Gossen (2006), using a microplate reader (Infinite M Flex, Tecan). Diverging from their protocol (which does not mention a filtration step), the solutions to measure Fluc and RLuc luminescence were filtered through a 0.45 μm filter (for *Renilla* measurement solution coelenterazine was added after the filtration step), the delay setting after each injection was 5 s (instead of 2 s), and the signal integration was 5 s (instead of 10 s). Three independent biological replicates with three technical replicates were performed. 1 µM GW7647 served as PC. FLuc luminescence was normalized to RLuc luminescence, PPARα activity was calculated relative to the SC.

### Benchmark dose modeling

The widely accepted BMD modelling approach was used to define BMD values for PPARα activation. It comprises a statistical methodology that assumes one or more underlying models to describe the dose-response relationship. The model(s) are fitted to the dose-reponse data to calibrate the model parameters (Davis et al. 2011; EFSA 2022). The BMR is defined as the degree of change defining a response that is measurable and reflects an adverse effect. It depends on the selected endpoint. The BMD is the dose or concentration that produces this predetermined BMR (EFSA 2022; Haber et al. 2018). The lower and upper 95% confidence intervals of the BMD method are termed benchmark dose lower bound (BMDL) and benchmark dose upper bound (BMDU). The ratio BMDU/BMDL can be used to characterize the uncertainty in BMD estimation. Thereby higher ratio values indicate greater uncertainty. The latest EFSA guidance document recommends the use of model averaging to estimate the BMD and its confidence interval. Therefore, we employed Bayesian model averaging to estimate the posterior distribution of the BMD, essentially treating BMD as a random variable. In accordance with EFSA’s latest guidance, eight continuous dose-response models were fitted to the data, each under both normal and log-normal distributional assumptions for the response variable, yielding a total of 16 candidate models. The specific models included exponential, inverse exponential, Hill, log-normal, gamma, quadratic exponential, probit, and logit functions (EFSA 2022). BMD estimation was conducted through Markov Chain Monte Carlo (MCMC) sampling. In total, 20,000 samples were drawn, with the first 10,000 iterations being considered warmup and, thus, discarded. The calculations were performed using a BMR of 1.5 relative to the SC.

### Quantitative structure-activity relationship (QSAR) model development

QSAR modelling was employed to map specific physicochemical properties of PFAS to the studied biological activity, in this case PPARα activation. Instead of modelling the raw concentration-response data directly, we utilized the BMD estimates as a compact representation of PPARα activation potency. Therefore, the model’s input included physicochemical properties of each congener, and the predicted response was the estimated BMD value.

A comprehensive set of SMILES-based descriptors was used to characterize each compound. In particular, the full design matrix was constructed by computing and combining the molecular access system (MACCS) fingerprints, extended connectivity fingerprints (ECFPs), Mordred, and RDKit descriptors for all congeners in the panel of selected chemicals. The MACCS fingerprints comprise a predefined set of 166 binary bits that encode the presence of specific molecular substructures through bit activation (1 if present, 0 if not present) (Durant et al. 2002). Similarly, ECFP fingerprints are circular topological fingerprints that extract information from atomic neighbourhoods and encode molecular substructures, though unlike MACCS fingerprints, these substructures are not predefined (Rogers and Hahn 2010). Both Mordred and RDKit descriptors consist of large sets of continuous features that include, among others, physicochemical, topological, and electronic descriptors, with the former providing approximately 1,800 descriptors and the latter around 200 (Moriwaki et al. 2018; RDKit 2025). In cases where salts were present, the SMILES representation of the salt form was used.

The goal of QSAR model development often lies on a spectrum between models optimized for high predictive performance, which are validated through various methodologies and metrics, and those focused on interpretability, or ideally, achieving both. However, models with strong predictive power typically require large and diverse datasets that span a broad chemical space, including representatives from multiple chemical classes. In this study, the dataset comprised 34 chemicals from five distinct PFAS subgroups. Given this limited and relatively focused dataset, we prioritized model interpretability. To that end, we selected a linear model architecture, offering a parsimonious and effective framework for uncovering meaningful patterns in the data. This approach allows straightforward interpretation of feature importance through the magnitude and sign of the model coefficients.

Feature preprocessing included data cleaning, dimensionality reduction, and normalization. Starting from the initial dataset containing descriptors from all four types (i.e., MACCS, ECFPs, Mordred, RDKit), the first step involved removing non-finite values, followed by the application of a low-variance filter to eliminate features with minimal informational content. Dimensionality was further reduced by removing highly correlated features. Specifically, in each pair of features with a Pearson correlation coefficient exceeding 0.9, one feature was discarded. The remaining features were then standardized.

The core focus of the model development pipeline was on feature selection, which was implemented using a genetic algorithm. It operates on an evolving population of solutions, where each individual represents a binary chromosome encoding the inclusion or exclusion of specific features. Individuals achieving higher scores in the objective function have a greater likelihood of passing their genetic material, i.e. the chromosome encoding selected features, to the next generation. The aim of the algorithm is to identify feature subsets that maximize model performance during cross-validation, while minimizing the number of selected descriptors. Different types of mutation, crossover, and selection strategies were tested, including uniform and two-point crossover, bit-flip and Gaussian mutation, as well as roulette wheel and tournament selection. The fitness of each individual was defined as the negative mean absolute percentage error (MAPE) of a linear model fitted via 5-fold cross-validation, adjusted by a regularization term proportional to the number of selected features, effectively penalizing for excessive feature inclusion in order to promote model parsimony. Cross-validation splits were re-randomized for each individual in the population to avoid overfitting to any particular split. To improve computational efficiency, model evaluation was parallelized across 10 Central Processing Unit (CPU) cores. The top three individuals from each generation were preserved to ensure that the best-performing feature subsets were retained throughout the optimization process.

Given the limited number of compounds in the dataset, splitting the data into independent training and test sets was not feasible. Instead, model performance was evaluated using 5-fold cross-validation. This approach allowed for a more robust assessment of generalizability, as a greater number of congeners were excluded from the training set and used for testing in each fold, thus providing a more realistic estimate of predictive performance. In addition to goodness-of-fit metrics obtained during training and cross-validation, y-randomization was applied to confirm that any observed structure-activity relationships were not the result of chance correlations.

An essential component of QSAR modelling is the AD, which assesses the reliability of predictions for new compounds. The AD defines the chemical space covered by the training data and determines whether a query compound is structurally similar enough for the model’s prediction to be considered trustworthy. Several methodologies exist for defining the AD of QSAR models (Sahigara et al. 2012). In this study, we employed a composite approach combining the bounding box and leverage methods. The bounding box method considers a compound within the AD if all its feature values fall within the range defined by the minimum and maximum values observed in the training set. The leverage approach, on the other hand, evaluates how far a compound lies from the centroid of the training data in descriptor space. For a given design matrix X, the leverage value h_i_ of an instance i is computed as:

$${\mathrm{h}}_{{\mathrm{i}}} = {\mathrm{x}}_{{\mathrm{i}}} ^{{\mathrm{T}}} \left( {{\mathrm{X}}^{{\mathrm{T}}} {\mathrm{X}}} \right)^{{ - {\mathrm{1}}}} {\mathrm{x}}_{{\mathrm{i}}}$$

where x_i_ is the descriptor vector of compound i. A prediction is considered outside the AD of the model, if h > h*, with the critical leverage h* defined as:

$${\mathrm{h}}* = {\mathrm{3}}\left( {{\mathrm{p}} + {\mathrm{1}}} \right)/{\mathrm{n}}$$

where p is the number of descriptors in the design matrix and n is the number of training compounds (Sahigara et al. 2012). In the present study, for a compound to be considered within the AD of the model only if its selected descriptor values satisfied the criteria of inclusion for both the bounding box and the leverage method.

### Software

BMD with Bayesian model averaging analysis was performed in R v.4.4.1 (R-Project 2021) through the ToxicR package (Wheeler et al. 2023). All plots were created using the ggplot2 R library (Wickham 2016).

QSAR modelling was implemented in Python v.3.10 (van Rossum 1995) using scikit-learn (Pedregosa et al. 2011) through the jaqpotpy package (https://jaqpot.org/docs). MACCS fingerprints, ECFP fingerprints and RDKit descriptors were generated using RDKit (RDKit 2025), Mordred descriptors were computed by the Mordred Python package (Moriwaki et al. 2018), and all descriptor generation tasks were facilitated by appropriate wrappers contained in jaqpotpy. Feature selection using genetic algorithms was implemented using Python package DEAP (Rainville et al. 2012).

## Results

### Cytotoxicity

**Fig. 1:**
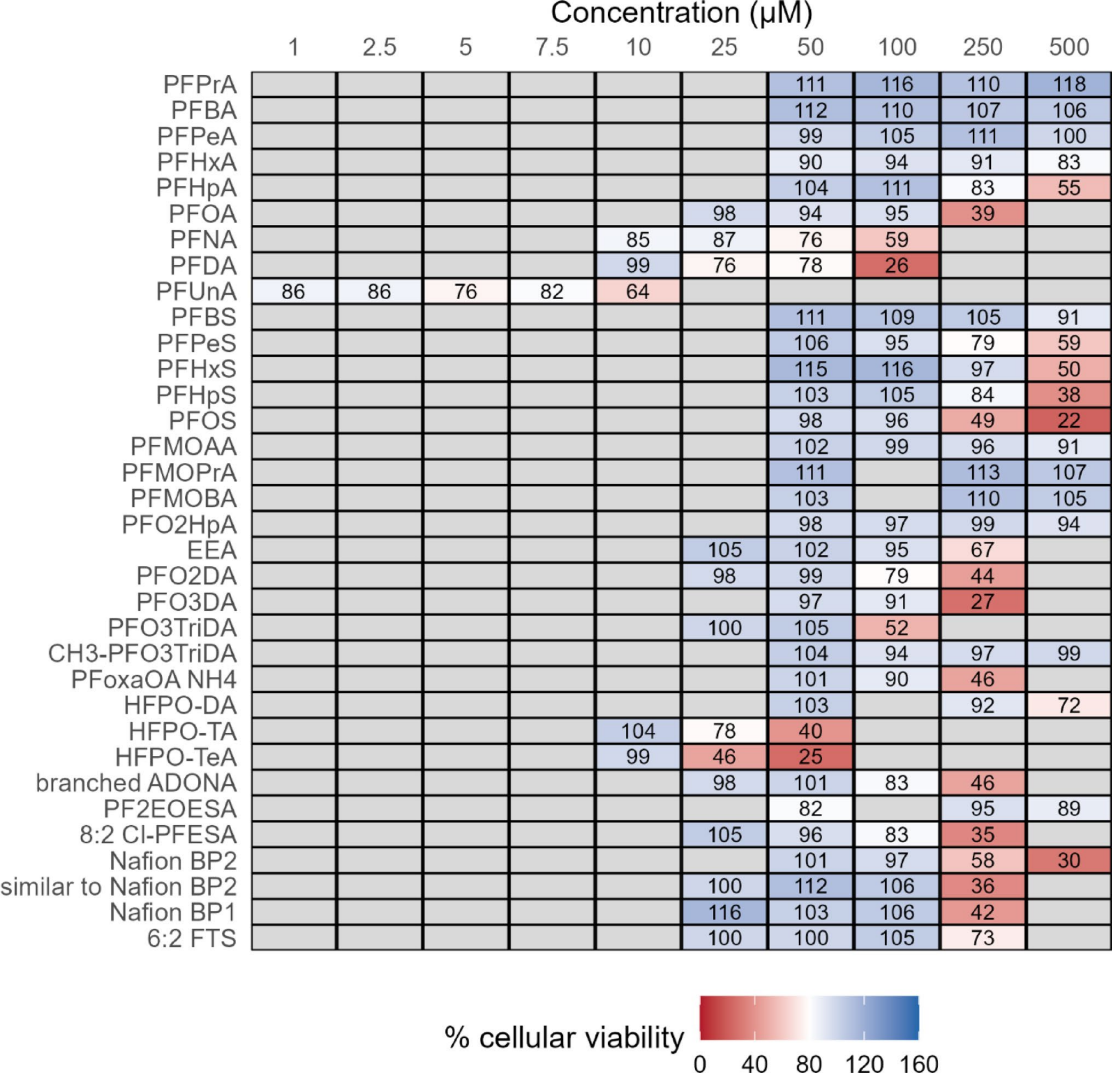
PFAS cytotoxicity in HEK293T cells. HEK293T cells were incubated with PFAS congeners and concentrations as indicated in the figure for 24 h before performing an MTT-assay. Values were normalized to untreated solvent control. Data show mean of three independent biological replicates with each three technical replicates. In the heatmap, cytotoxicity is illustrated by red color indicating that cellular viability was below the defined threshold of 80%. Detailed cytotoxicity data are given in Supplementary Fig. 1A–D

To define the concentration range for the reporter gene experiments, potential cytotoxic effects of the 34 different PFAS on HEK293T cells had to be determined first. Cells were incubated with different concentrations of the 34 PFAS for 24 h, and cellular viability was evaluated by means of the MTT assay. The results are given in Supplementary Fig. 1A–D and are summarized as a heatmap in Fig. 1. A PFAS concentration was defined to be cytotoxic when the corresponding MTT value was either less than 80% relative to the value of the untreated negative control or at least 10% below the value of the next smaller test concentration. A number of PFAS did not induce cytotoxic effects in HEK293T cells up to the highest test concentration of 500 µM; these were the short-chain PFAS PFPrA, PFBA, PFPeA, PFMOAA, PFMOPrA, PFMOBA, PFO2HpA and PF2EOESA and the methylated congener CH3-PFO3TriDA. PFBS, PFHxA and HFPO-DA were not cytotoxic up to a concentration of 250 µM, PFHpA, PFOA, EEA, PFPeS, PFHxS, PFHpS, PFOS, PFO3DA; Nafion-BP2, similar to Nafion-BP2, Nafion-BP1 and 6:2 FTS were not cytotoxic up to a concentration of 100 µM, PFO2DA, PFO3TriDA, PFoxaOA, branched ADONA and 8:2 Cl-PFESA were not cytotoxic up to a concentration of 50 µM, and PFNA, PFDA, HFPO-TA and HFPO-TeA were not cytotoxic up to a concentration of 10 µM. For PFUnA, viability of HEK293T cells was reduced to a level of about 80% for all tested concentrations.

### PPARα induction

A PPARɑ-dependent reporter gene assay was applied to examine the potential of the 34 PFAS congeners to activate PPARɑ. HEK293T cells were transfected with the plasmids required to conduct the luciferase-based reporter gene assay and subsequently incubated with the 34 PFAS for 24 h. At least seven different test concentrations were chosen for each PFAS, covering a range from 0.1 µM up to the respective highest non-cytotoxic concentration as determined in the MTT assays (see above). The results of the reporter gene assays are given in Supplementary Fig. 2A–D. All PFAS examined in the present study were capable of activating PPARɑ in a concentration-dependent manner except for PFDA, PFUnA and 8:2 Cl-PFESA which did not activate PPARɑ up to the highest test concentration (Supplementary Fig. 2A–D).

**Fig. 2:**
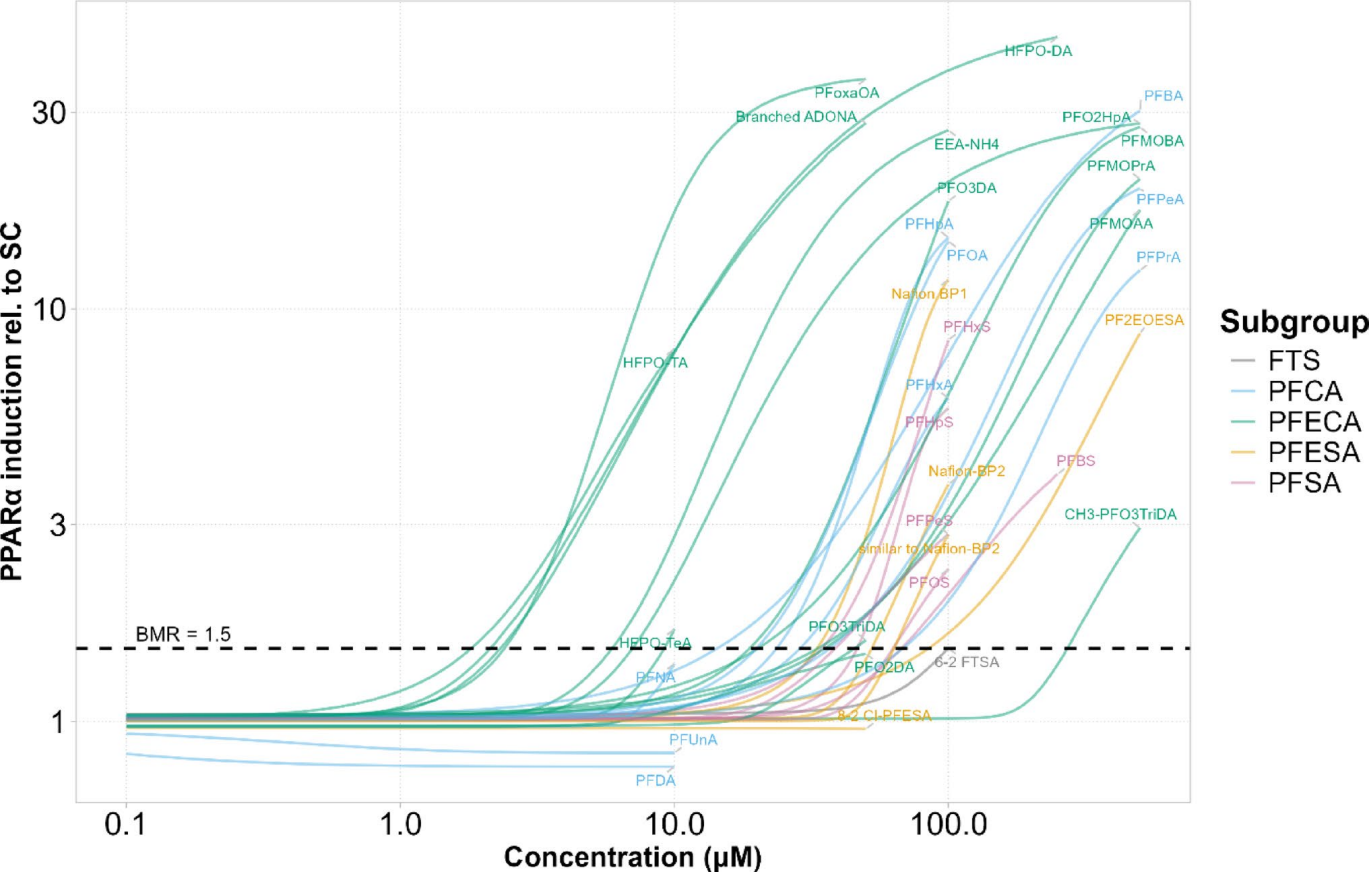
Concentration-response curves for PPARα activation, generated through Bayesian model averaging. The response represents induction relative to the solvent control (SC). Each curve corresponds to the median fit derived from Markov Chain Monte Carlo (MCMC) simulations. PFAS subgroups are distinguished by color: blue for perfluoroalkylcarboxylic acids (PFCA), green for perfluoroalkylether carboxylic acids (PFECA), orange for perfluoroalkylether sulfonic acids (PFESA), black for fluorotelomer sulfonates (FTS), and pink for perfluoroalkyl sulfonic acids (PFSA)

BMD modeling was applied to the concentration-response data to compare the PPARɑ activation potency of the 34 PFAS. Supplementary Table 2 presents the estimated BMD, along with the BMDL and BMDU for each of the tested PFAS. For PFDA, PFUnA, 8:2 Cl-PFESA and 6:2 FTS, no statistically significant ascending relationship between concentration and response was observed within the tested concentration range, and therefore BMD estimation was not feasible. Among the remaining congeners, PFO2DA showed particularly high uncertainty in its estimate, as indicated by a large ratio between the upper and lower BMD bounds. Figure 2 presents the concentration-response curves generated using the Bayesian model averaging framework, with each curve representing the median fit derived from MCMC sampling. The plot highlights that certain PFECA, such as HFPO-DA and branched ADONA, exhibit notably higher potency to activate PPARɑ than other congeners. No clear pattern emerges from the plot with respect to maximum activation, primarily because the highest tested concentration for many congeners was limited by cytotoxicity constraints. As a result, several concentration-response curves do not reach a plateau, making it difficult to draw definitive conclusions regarding maximal activation.

**Fig. 3:**
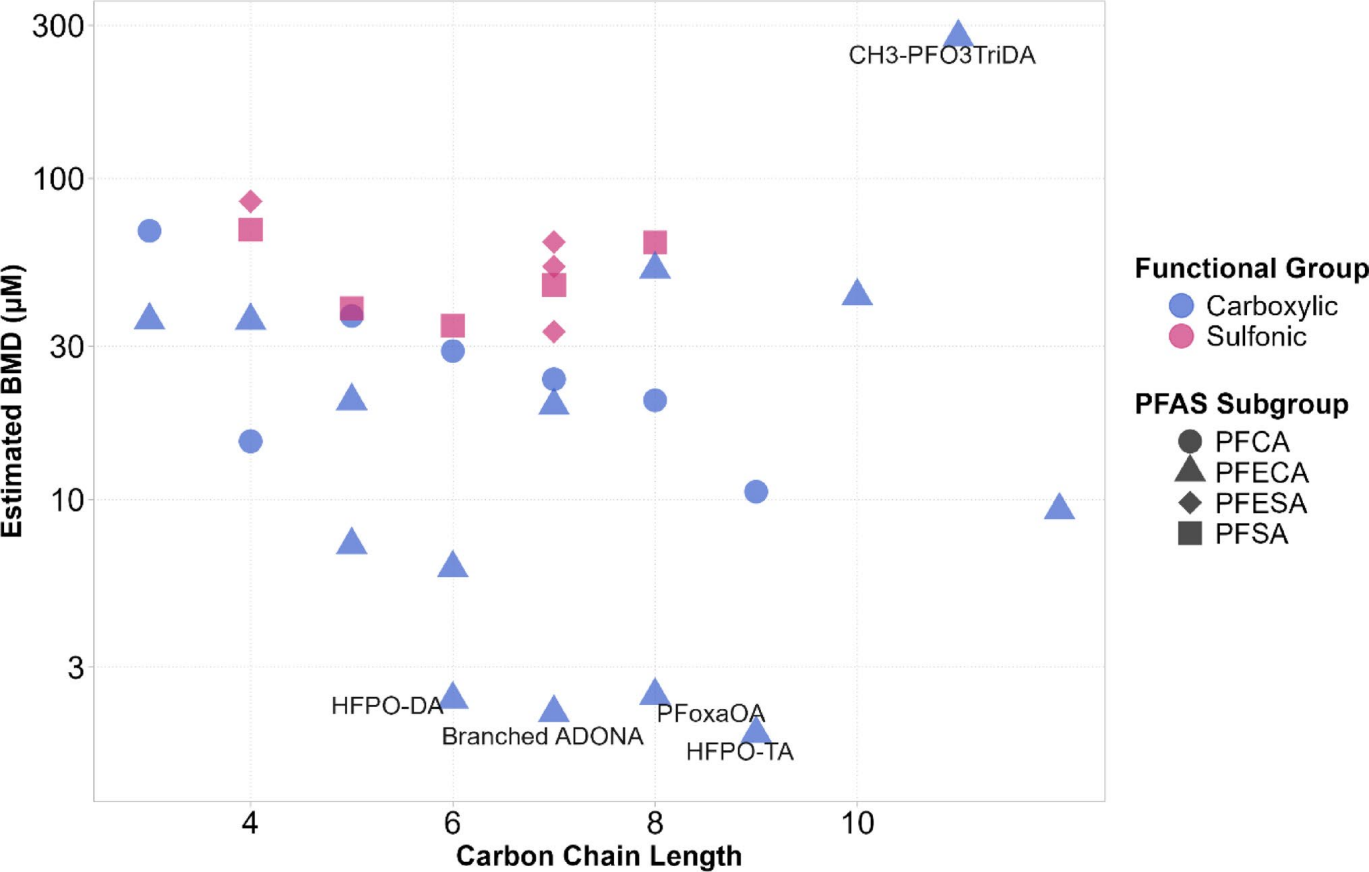
Scatter plot of benchmark dose (BMD) estimates for a benchmark response (BMR) of 1.5 plotted against carbon chain length for the tested PFAS. Compounds containing a carboxylic group are shown in blue, and those containing a sulfonic group are shown in pink. PFAS subgroups are differentiated by marker shape: perfluoroalkylcarboxylic acids (PFCA) as circles, perfluoroalkylether carboxylic acids (PFECA) as triangles, perfluoroalkylether sulfonic acids (PFESA) as rhombuses, and perfluoroalkyl sulfonic acids (PFSA) as squares

Figure 3 shows the BMD estimates plotted against carbon chain length, with color used to distinguish between PFAS containing a sulfonic group or a carboxylic group and shape representing the five PFAS subgroups studied: PFCA, PFSA, PFECA, PFESA and FTS. While no consistent linear association was observed between carbon chain length and the estimated BMD values, a clear trend emerged depending on the functional group of the compounds. Those with a sulfonic group tended to display higher BMD values and thereby a lower potency to activate PPARα compared to those with a carboxylic group. When the number of oxygen atoms in each structure was encoded with color, no distinct pattern with respect to potency could be detected. Similarly, no relationship was found between molecular branching and the estimated BMD. One molecule, CH3-PFO3TriDA, was identified as an outlier with a notably high BMD estimate, indicating very low potency. This might be due to the fact that this is the only congener within the set of the 34 PFAS in which the carboxylic function is methylated. In contrast, the most potent inducers in the dataset were HFPO-DA, branched ADONA, HFPO-TA, and PFoxaOA, all of which belong to the PFECA subgroup. These four compounds exhibited BMD values below 3 µM.

**Fig. 4:**
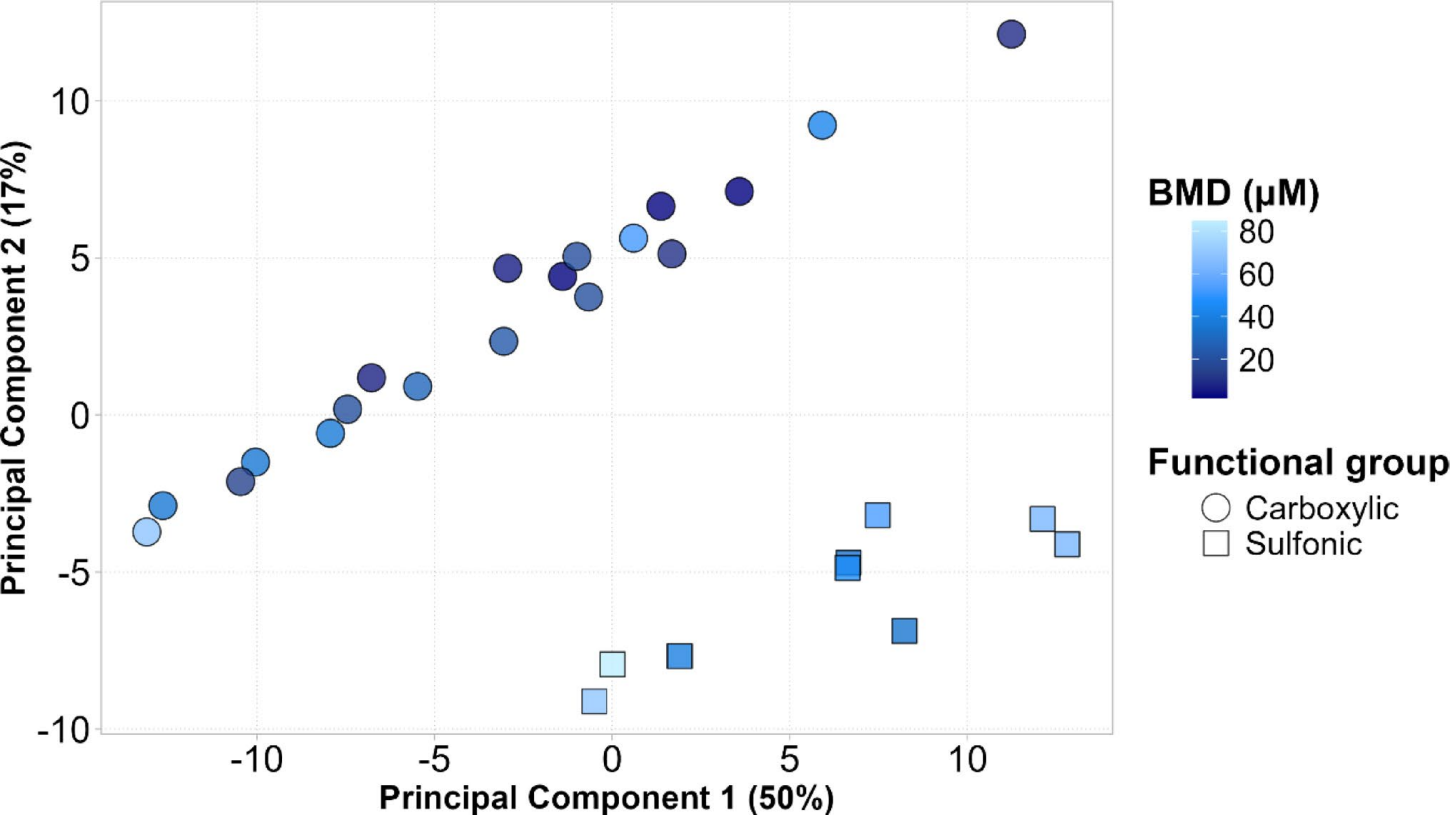
Principal component analysis (PCA) plot of the first two components derived from Molecular ACCess System (MACCS) fingerprints and RDKit descriptors. Each point represents a PFAS, with color intensity encoding the benchmark dose (BMD) estimate. Lighter shades correspond to higher BMD values, indicating lower potency for PPARα induction. The percentage of variance explained by each principal component is shown in parentheses on the corresponding axes. The figure displays only those PFAS for which BMD values could be estimated

To further explore PPARα activation, BMD results were integrated with principal component analysis (PCA) to generate informative visualizations and identify patterns linking the physicochemical properties of PFAS to their observed potential to activate PPARα in vitro. PCA was conducted both on the complete descriptor set, consisting of the MACCS fingerprints, ECFP fingerprints, Mordred and RDKit descriptors, and separately on each individual descriptor group. The most interpretable and informative visualizations were obtained when using the MACCS fingerprints along with the RDKit descriptor as input. These results are presented in Fig. 4. The trend observed in Fig. 3, which suggested that the presence of a carboxylic group results in a higher potency to activate PPARα compared to PFAS with a sulfonic group becomes even more pronounced in the principal component space. A clear separation is observed between PFAS compounds containing a carboxylic group and a sulfonic group, driven primarily by the first principal component, which explains 50% of the variance in the dataset. PFAS with a sulfonic group cluster together and are generally associated with lighter shades of blue in the plot, indicating higher BMD values and therefore lower potency for PPARα activation. At the same time PFAS with a carboxylic group are associated with darker blue, indicating lower BMD values and therefore higher potency for PPARα activation. PCA loadings, which represent the contribution of each fingerprint variable to a principal component, are commonly used to identify which features drive the separation between samples. In this case, examination of the loadings associated with the first principal component, which drives the separation of PFAS with a carboxylic group and a sulfonic group, did not identify a single fingerprint as dominant. However, most of the ten most influential fingerprints contributing to the first principal component involved features related to sulfur and oxygen-containing groups or bonding patterns. These include, for example, fingerprint f88, which encodes the presence of a sulfur atom, f136, which represents an oxygen atom double bonded to any atom, f40, which captures an S-O bond, and f73, which corresponds to a sulfur atom double bonded to any atom.

### QSAR modelling

**Fig. 5:**
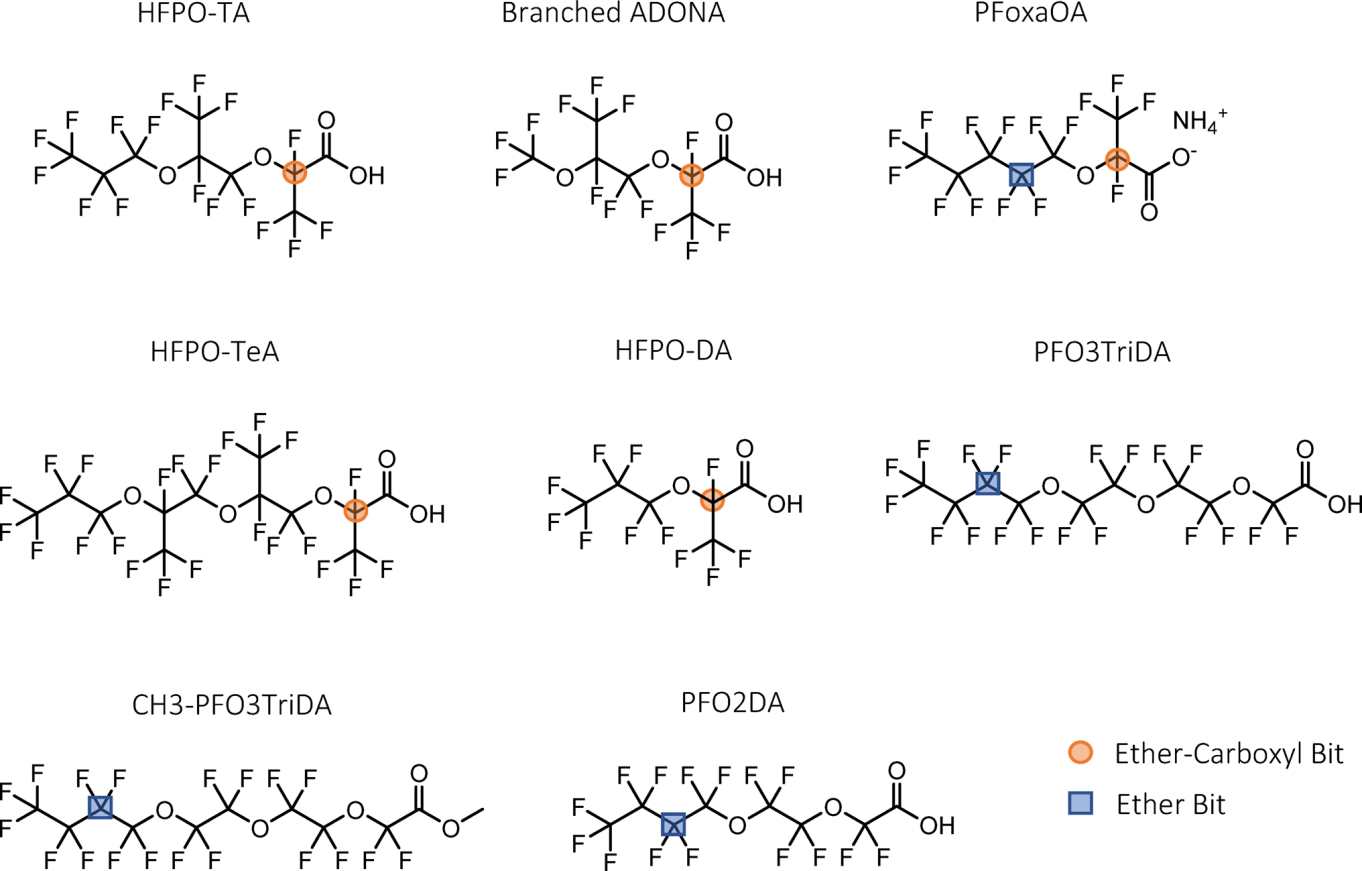
Molecular structures of the tested PFAS that comprise key substructural features used in the QSAR model. The Ether Bit (blue square) is represented by a carbon atom in the center of a linear R-CF2-CF2-CF2-CF2-O-R moiety. The Ether-Carboxyl Bit (red circle) is present when a carbon is bonded to a carboxyl group, an ether oxygen, a fluorine, and a fluorinated carbon. Both features are derived from extended-connectivity fingerprints (ECFP) and were part of the final model’s feature space. PFoxaOA is the only congener of the set of 34 PFAS that comprises both features

The first step in the predictive modelling pipeline involved the computational generation of molecular descriptors from the SMILES representations of the tested congeners. Following the removal of columns containing non-finite values, the combined descriptor set included approximately 6,000 features. A low variance filter was applied, which eliminated around 4,700 features with limited informational content. Dimensionality was further reduced by removing highly correlated features, resulting in a final set of 141 descriptors available for feature selection. Feature selection using genetic algorithms utilized a population size of 300 and converged after approximately 30 generations. The optimal strategy consisted of tournament selection with a tournament size of 3, uniform crossover with a probability of 0.75, and bit-flip mutation applied with a probability of 0.25. This process selected a total of seven features, consisting of two binary fingerprints and five continuous descriptors. Based on the values of these seven features of a given substance, the developed QSAR model is able to predict the BMD of PPARα activation. The binary fingerprints were both ECFP bits, which represent specific substructures within the molecule but are not predefined, as is the case with MACCS fingerprints. These ECFPs were calculated using an atomic neighborhood radius of three, meaning each atom in the molecule was characterized based on its own properties as well as those of all atoms within three bonds. Both selected ECFP bits correspond to substructures involving ether groups. The first bit (in the following called Ether-Carboxyl Bit) is present when a carbon atom is bonded to a carboxyl group, an ether oxygen, a fluorine, and a fluorinated carbon. The second bit (in the following called Ether Bit) is represented by a carbon in the center of a R-CF2-CF2-**C**F2-CF2-O-R moiety (Fig. 5).

The selected continuous descriptors included AATS5dv, ATCS5dv, ATSC6c, and GATS5p from the Mordred descriptor set, as well as topological polar surface area (TPSA) from the RDKit descriptor set. None of these features exhibited strong correlation with other, more readily interpretable descriptors. ATCS5dv, AATS5dv, and ATSC6c are Broto-Moreau autocorrelation descriptors calculated at lag 5 (for ATCS5dv and AATS5dv) and lag 6 (for ATSC6c), respectively (Hollas 2003; Moreau and Broto 1980). These descriptors quantify the distribution of specific atomic properties across the molecular topology by evaluating all pairs of atoms separated by a fixed number of bonds, in this case by 5 bonds and 6 bonds, respectively. For each such pair, the property values of the atoms are multiplied and the resulting products are summed across the molecule. In ATCS5dv, the property used is the mean-centered valence electron count, in AATS5dv it is the raw valence electron count, which is then averaged by the total number of contributing pairs, and in ATSC6c it is the mean-centered Gasteiger-Marsili atomic partial charges (Gasteiger and Marsili 1980). GATS5p, in contrast, is a Geary’s autocorrelation descriptor computed at a topological distance of five bonds and weighted by atomic polarizability, a measure of how easily an atom’s electron cloud can be distorted by an external electric field (Choudhary et al. 2019; Geary 1954). These autocorrelation descriptors are useful for capturing topological and electronic patterns in a molecular structure, but are not easily interpretable. TPSA quantifies the surface area occupied by polar atoms, typically nitrogen and oxygen, and serves as a widely used predictor of membrane permeability, with higher TPSA values generally associated with reduced cellular uptake (Di and Kerns 2016; Prasanna and Doerksen 2009). Supplementary Table 3 presents the values of the seven descriptors for the studied 34 PFAS congeners. The sorting by functional group and increasing carbon chain length allows to explore the variation of these continuous descriptors depending on the structural features. Notably, both ATSC5dv and GATS5p increase consistently with PFCA chain length.

**Fig. 6:**
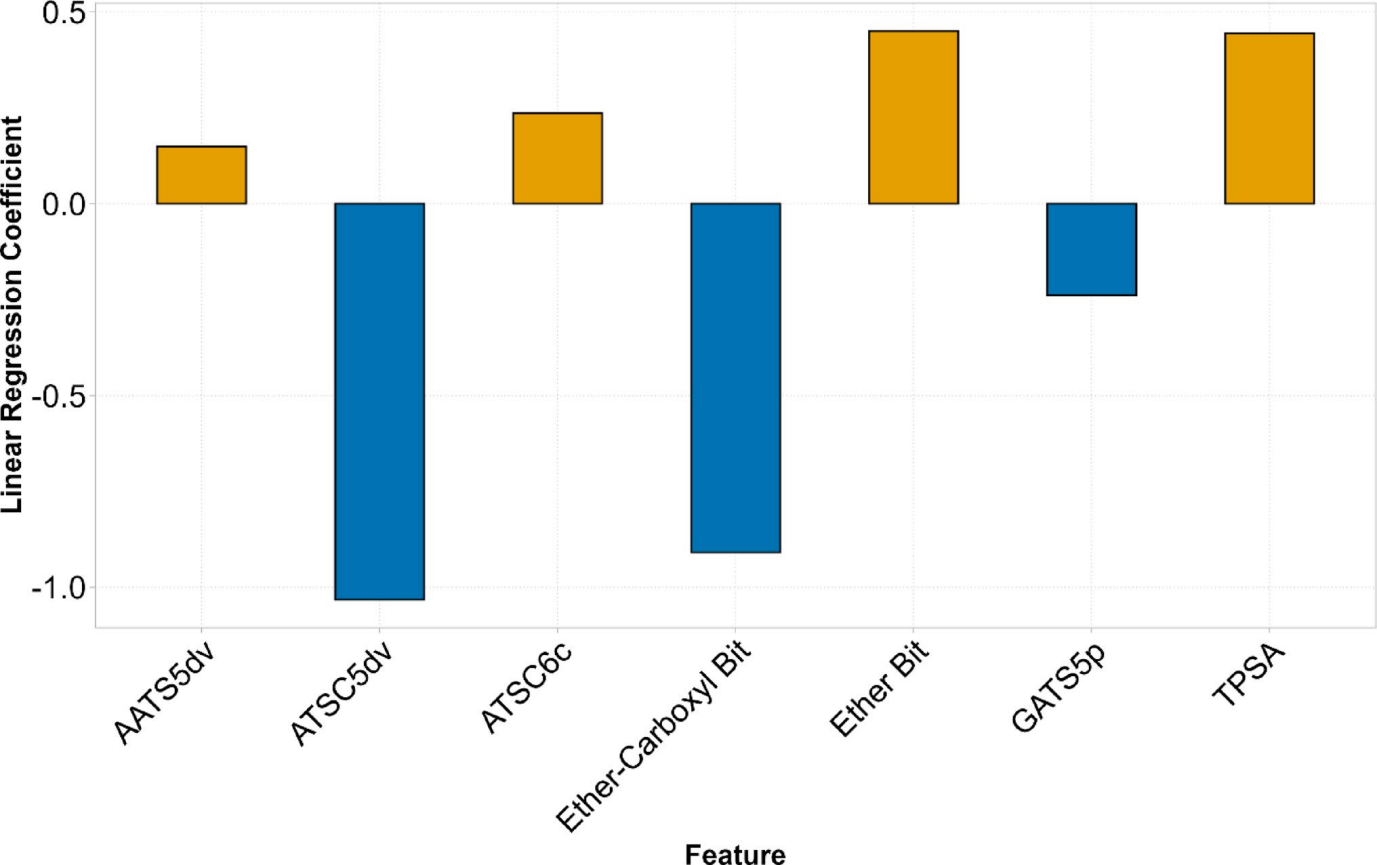
Coefficients of the linear model trained to predict BMD values. Each bar represents the weight assigned to a selected feature, with the sign and magnitude indicating the direction and strength of the feature’s contribution to PPARα induction potency. Positive coefficients (orange bars) are associated with increased BMD (lower potency) whereas negative coefficients (blue bars) correspond to decreased BMD (higher potency)

The final design matrix consisted of the seven selected features, which were used after standardization. Supplementary Table 3 presents the final design matrix along with the corresponding BMD value of each congener. To account for differences in response scale and reduce the influence of extreme values, a logarithmic transformation was applied to the response variable during modelling. However, all performance metrics were computed in the original, non-transformed scale, by inverse-transforming all predictions before comparing them to the respective observations. The coefficients of the fitted linear model are shown in Fig. 6. Based on the coefficient values, the two most influential descriptors were ATCS5dv and the Ether-Carboxyl Bit, both were associated with lower BMD values. According to the developed QSAR model, the presence of the Ether-Carboxyl substructure as well as high values of ATCS5dv induce an increased potency of PFAS to activate PPARα. This trend is in agreement with the pattern observed in Fig. 3. The presence of the Ether Bit substructure as well as high values of AATS5dv, ATSC6c, GATS5p and TPSA induce a decreased potency.

The metrics used to assess goodness-of-fit included the coefficient of determination (R^2^), mean absolute error (MAE), and root mean squared error (RMSE). As shown in Table 1, the model demonstrated strong performance in both training and 5-fold cross-validation. To confirm that the observed correlation between the selected descriptors and PPARα induction was not due to chance, 20 y-randomization tests were conducted. In these tests, the response variable was randomly shuffled and the model was re-fitted to the permuted data. The poor performance metrics observed during y-randomization, presented in Table 1, indicate that the relationship identified between the molecular descriptors and the biological response is statistically meaningful and not the result of random correlation.

**Table 1:** Performance metrics for the training, cross-validation, and y-randomization procedures. Reported metrics include the coefficient of determination (R^2^), mean absolute error (MAE), and root mean squared error (RMSE). For y-randomization, the values represent the mean performance across 20 repetitions, where the response variable was randomly permuted prior to model fitting, providing a baseline to assess the likelihood of chance correlations

	*R* ^2^	MAE	RMSE
Training	0.92	9.88	13.72
Cross-validation	0.84	13.95	19.72
Y-randomization	-688.51	23.87	29.93

**Fig. 7:**
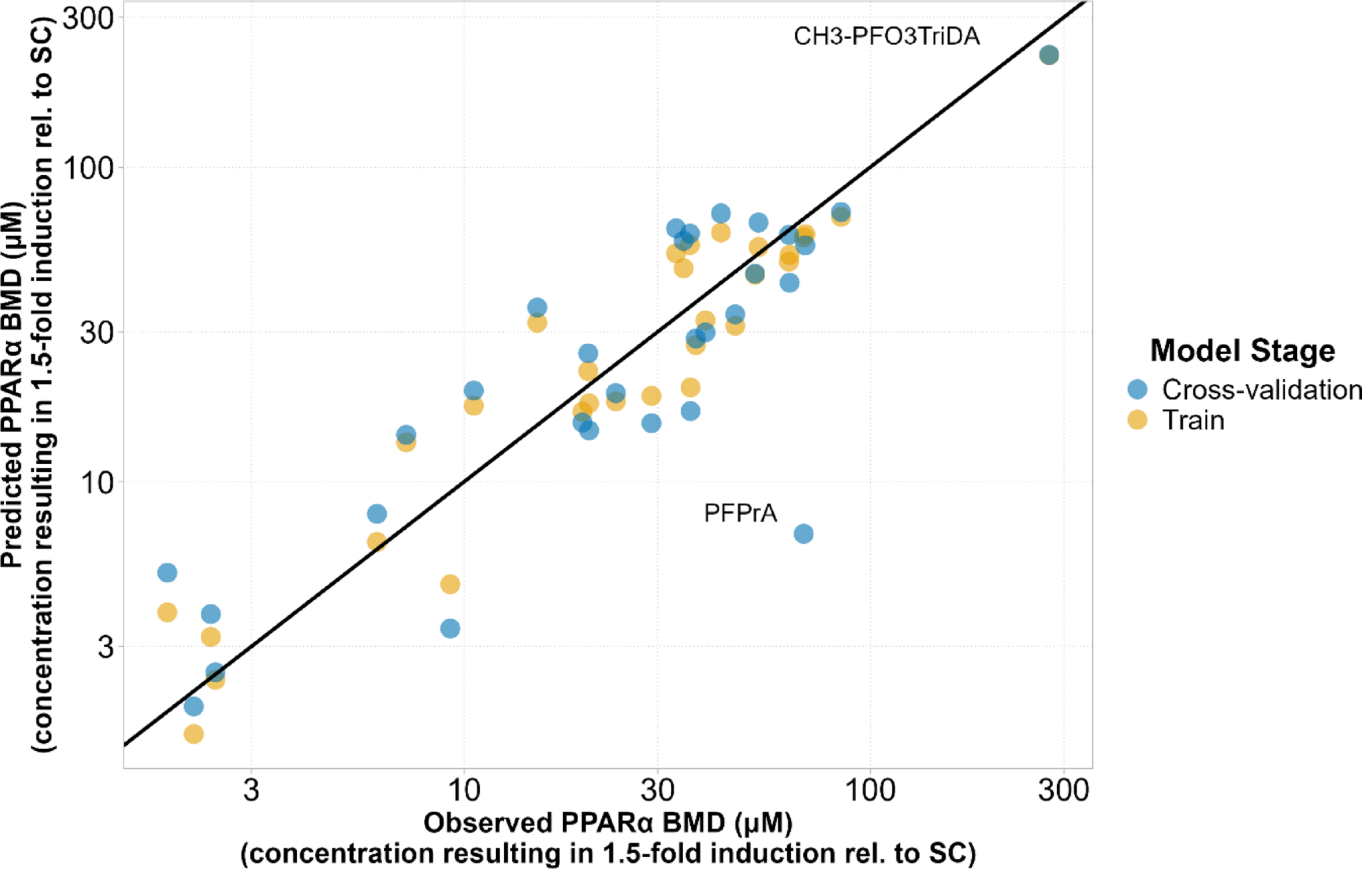
Predicted versus observed benchmark dose (BMD) values for model training (orange) and cross-validation (blue). The solid diagonal line represents the identity line (y = x), where predicted values exactly match the observed ones. Deviation from this line indicates the degree of prediction error, offering a visual assessment of model accuracy and generalizability. The BMD represents the concentration estimated to lead to a 1.5-fold induction of PPARα activity relative to solvent control (SC)

The predicted versus observed BMD values for both the training and cross-validation stages are shown in Fig. 7. For cross-validation, each point represents the model’s prediction in the fold where the corresponding PFAS congener was included in the test set. The majority of predictions fall close to the identity line, indicating strong model performance. The only clear deviation was observed for PFPrA, where two of the seven selected descriptors, specifically ATSC6c and AATS5dv, were reported as zero, unlike the corresponding values for the other congeners. This discrepancy likely arises from a limitation in descriptor calculation for this specific congener, given that PFPrA is structurally similar to the other PFCA compounds and such variation in descriptor values would otherwise be unexpected. As shown in Fig. 6, both of these descriptors contribute positively to the BMD value. Therefore, if PFPrA had descriptor values more consistent with the rest of the dataset, its predicted BMD would likely be higher and thus closer to the experimentally observed value. Supplementary Table 4 provides a detailed comparison of predicted and observed values during cross-validation. The table also includes applicability domain evaluations for the two methods used in this study. The congeners identified as lying outside the AD during cross validation, according to both the bounding box and leverage methods, were PFPrA, PFO3DA, CH_3_-PFO3TriDA, and EEA. Additionally, four congeners, in particular PFHxS, PFBS, PFoxaOA, and the compound similar to Nafion-BP2, were flagged as outside the domain by only one of the two methods. It is important to note that ADs were assessed based on the seven selected descriptors, rather than the full initial descriptor set.

**Fig. 8:**
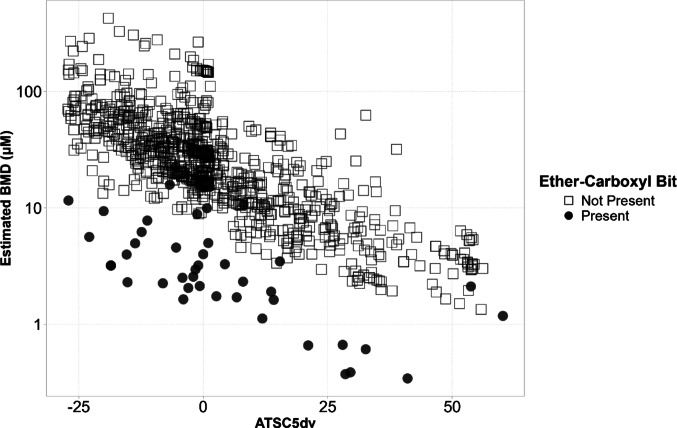
Screening plot of the 1,048 congeners from the PFASSTRUCTV4 list (Williams et al. 2022) that are within the described composite AD, displaying estimated benchmark dose (BMD) values (µM) against the ATSC5dv descriptor. Compounds are color-coded based on presence or absence of the “ether-carboxyl” ECFP fingerprint bit: filled circles (●) indicate presence (bit = 1), while open squares (□) represent absence (bit = 0). This visualization highlights how specific structural features relate to predicted potency, aiding the identification of potential strong PPARα inducers. Predictions outside the model’s applicability domain were excluded from the plot. The ten most potent compounds with the lowest predicted BMD values can be found in Table 2

Following evaluation, the model was applied to screen the PFASSTRUCTV4 list, which includes 10,776 PFAS congeners (Williams et al. 2022). The goal was to map the chemical landscape of PFAS with respect to their potential to induce PPARα and to identify strong candidate inducers. During this screening phase, the AD served to distinguish reliable predictions from those that should be treated with caution due to insufficient structural similarity of the screened PFAS congeners with the training compounds. Of the screened chemicals, 1,048 compounds, roughly 10% of the total, were found to fall within the boundaries of the described composite AD. These compounds were visualized in relation to the two most influential descriptors, ATSC5dv and the Ether-Carboxyl Bit. As illustrated in Fig. 8, the most potent predicted compounds were those that have the Ether-Carboxyl Bit present in their molecular structure. Among the predicted BMD values, the median was 20 µM. Some of the predicted BMD values are as low as below 1 µM.

**Table 2:** Top ten PFAS with the highest predicted PPARɑ activation potency (lowest BMD values). Further information on these PFAS congeners can be obtained via the DTXSID identifiers (https://comptox.epa.gov/dashboard/)

No. (see Fig. 8)	Name	Predicted BMD (µM)	DTXSID
1	8-Methylperfluoro-3,6-dioxa-2,5-dimethyloctanoic acid	0.34	DTXSID201023778
2	Methyl 2,3,3,3-tetrafluoro-2-[1,1,2,3,3,3-hexafluoro-2-[(1,1,2-trifluoro-2-propen-1-yl)oxy]propoxy]-propanoate	0.37	DTXSID301023719
3	Methyl perfluoro-2-[2-(3,3,3-trichloropropoxy)propoxy]-propanoate	0.39	DTXSID901023729
4	Ethyl-2,4,4,5,7,7,8,10,10,11,13,13,14,16,16,17,19,19,20,20, 21,21,21-tricosafluoro-2,5,8,11,14,17-hexakis(trifluoromethyl)-3,6,9,12,15,18-hexaoxahenicosan-1-oate	0.61	DTXSID70896538
5	5-Methylperfluoro-3-oxa2-methylpentanoic acid	0.66	DTXSID501023777
6	2,3,3,3-Tetrafluoro-2-(1,1,2,3,3,3-hexafluoro-2-((1,1,2-trifluoro-2-propen-1-yl)oxy)propoxy)propanoic acid	0.67	DTXSID601023720
7	2,3,3,3-Tetrafluoro-2-[(1,1,2-trifluoro-2-propenyl)oxy]-propanoate	1.12	DTXSID501023589
8	Ammonium 2,3,3,3-tetrafluoro-2-(nonafluorobutoxy)propanoate	1.18	DTXSID10896638
9	2,2-Dichloro-perfluoro-1-butanesulfonic acid	1.35	DTXSID401035264
10	11:2 Fluorotelomer sulfonic acid	1.51	DTXSID801009441

The QSAR model reporting format (QMRF) QMRF is provided in Supplementary Table 5. The QSAR model has been deployed on the Jaqpot platform as a web service and is accessible at the following link: https://app.jaqpot.org/dashboard/models/2058/description%22%20/h.

## Discussion

PFAS-mediated PPARα activation has been demonstrated for several PFAS in vitro (Barutcu et al. 2024; Behr et al. 2020; Bjork and Wallace 2009; Buhrke et al. 2015; Evans et al. 2022; Houck et al. 2021; Rosenmai et al. 2018; Wolf et al. 2008), and it is well accepted that the impact of PFAS on PPARα activity may be an important driver for PFAS toxicity, at least in rodents (Kudo et al. 2000; NTP 2019a, b). PPARα is a central regulator of hepatic lipid metabolism, and alterations of PPARα activity are typically associated with perturbations in lipid homoeostasis that may lead in vivo to various adverse outcomes such as liver steatosis or cholestasis. In rodents, excessive PPARα activation may even lead to hepatocellular adenomas and carcinomas as outlined in AOP #37 (Corton 2023). In this AOP, PFOA is listed among others as a typical stressor activating this specific AOP via PPARα. Notably, AOP #529 was created to specifically describe the PFOS-mediated dysregulation of lipid metabolism and subsequent liver steatosis, again via PPARα activation (Mylroie et al. 2025). Taken together, these findings highlight PPARα as a key molecular target of PFAS. However, given the existence of more than 10,000 PFAS congeners (Williams et al. 2022), it is not feasible to experimentally characterize each of them in detail. Therefore, in this work we aimed to develop a model capable of predicting the potency of PFAS to activate PPARα, assuming that PPARα activation can serve as an alert for potential PFAS toxicity.

In this study, we experimentally determined the capacity of 34 different PFAS congeners to activate PPARα by means of a well-established PPARα-dependent reporter gene assay. In addition to several congeners from the classic PFCA and PFSA subgroups, particular emphasis was placed on the PFECA and PFESA subgroups of (poly)-ether PFAS, as these compounds have been developed as replacements for substances such as PFOA and PFOS in numerous industrial applications. Consequently, these replacement PFAS have emerged as environmental contaminants, yet their potential impact on human health remains largely unknown.

For many congeners, PFAS cytotoxicity prevented from reaching a plateau for maximum activation in the concentration-response curves (see Fig. 2). The maximum activation observed for those congeners for which the concentration-response curve reached the plateau was in the range of 30-fold induction. This was in the same range as observed for the positive control GW7647, indicating that PFAS are as potent PPARα agonists as synthetic PPARα agonists, at least in terms of maximal activation. We preferred not to use the maximal activation, but instead used a BMR as of point of departure to access PPARα activation potencies of PFAS. To our knowledge, no biologically relevant BMR for in vitro or in vivo has been established for a PPARα transactivation assay. In several Test Guidelines (TG) by the Organisation for Economic Co-operation and Development (OECD), a 1.5-fold induction of the respective biological test system has been defined as the threshold for a positive classification. As an example, OECD TG 442D is also based on a luciferase reporter gene assay and uses a 1.5-fold induction as a threshold for a positive result (OECD 2024a). Other OECD TGs, e.g., TG 456 and TG 442E, also use a 1.5-fold induction relative to the control as a threshold for positive classification (OECD 2024b; OECD 2025). Based on these TGs and on the variance between biological replicates, we set the BMR to 1.5. Although no conclusion can be drawn as to whether a 1.5-fold induction in vitro indicates an adverse in vivo effect, this threshold appeared more meaningful than the EFSA default of 1.05 given that some PFAS induced up to 40-fold activation (EFSA 2022).

Using the 1.5-fold induction as threshold, a BMD was derived for all PFAS congeners examined in the present study except for PFDA, PFUnA, 8:2 Cl-PFESA and 6:2 FTS. Notably, the lowest BMD values in the range of 1–3 µM were obtained for some members of the PFECA subgroup (HFPO-DA, HFPO-TA, branched ADONA and PFoxaOA), indicating that these congeners are more potent in PPARα activation compared to the members of the PFCA subgroup (Figs. 2 and 3). Moreover, our analysis showed that PFAS with a sulfonic acid functional group seem to be less potent in PPARα activation than PFAS congeners with a carboxylic acid (Figs. 2 and 3). Overall, our results are in good agreement with previous reported PPARα activation data from our own lab for PFOA, PFOS, PFHxS, PFNA, PFHxA, PFBA, PFBS and HFPO-DA, showing good reproducibility of the data when the same test system is used (Behr et al. 2020; Sadrabadi et al. 2023). By using similar but not identical PPARα-dependent reporter gene assays, Wolf et al. investigated PPARα activation in COS-1 cells transiently transfected with plasmids expressing either human or mouse PPARα (Wolf et al. 2008, 2012). In agreement with our findings, they observed that PFSA were less potent than PFCA, and that PFDA did not activate the receptor. Rosenmai et al. employed a luciferase reporter gene assay in HepG2 cells and directly quantified intracellular PFAS levels to relate internal exposure to PPARα activation. They reported significant activation for all tested congeners except PFOS, PFUnDA, and PFOSA, with PFOA inducing the highest response. Comparisons between PFCA and PFSA of the same chain length again showed lower potency for PFSA, consistent with our observations (Rosenmai et al. 2018). In the case of PFOS, lower PPARα activation compared to the other tested PFAS could be attributed to limited cellular uptake. More recently, Evans et al. used a luciferase reporter gene assay based on a commercial, non-human cell line overexpressing PPARα. Their study identified HFPO-DA being a more potent congener compared to legacy PFAS, aligning with our results (Evans et al. 2022). Among nearly all in vitro studies published so far, there is a consensus that HFPO-DA is a strong activator of PPARα. Transcriptomic analyses of rat and human hepatocytes following HFPO-DA exposure suggest that its mode of action involves PPARα (Heintz et al. 2024a, b). There is also in vivo evidence of adverse effects in rodents after HFPO-DA (GenX) exposure, with activation of PPARα-related pathways (Conley et al. 2019). A recent study further showed that the disruption of hepatic lipid metabolism in mice by HFPO-DA (GenX) was induced by PPARα activation (Attema et al. 2022). Another study reported that rodent exposure to several HFPO-DA (GenX) analogues, including branched ADONA, resulted in even greater liver toxicity than HFPO-DA itself through PPARα-dependent mechanisms (Ren et al. 2024). Branched ADONA and HFPO-DA belong to the most potent PPARα agonists in our study, supporting the notion that PPARα activation potency might correlate with adverse effects in vivo.

Notably, 31 of the 34 PFAS congeners examined, were capable of activating human PPARα in vitro. Only three congeners (PFDA, PFUnA and 8:2 Cl-PFESA) did not activate PPARα in our assay. However, this lack of activity is likely attributable to cytotoxicity constraints, which prevented these PFAS from being tested at concentrations comparable to those used for the other tested congeners.

The experimental data obtained for the 34 PFAS congeners were used for BMD analysis and for building a QSAR model to identify substructures and physicochemical properties that drive the observed in vitro PPARα activation and to predict BMD values for PPARα activation. Feature selection using a genetic algorithm combined with a simple linear model enabled robust dimensionality reduction and led to the development of a parsimonious model with only seven features retained. We assumed linear correlation between the computational features and BMD to keep the modeling straightforward and the results easily interpretable. However, we cannot exclude the possibility that nonlinear relationships might be the main drivers of the observed responses. The genetic algorithm was preferred over other feature selection methods such as recursive feature elimination, since the latter can discard valuable features early in the process due to the high noise associated with high-dimensional data. In contrast, genetic algorithms are better at capturing interactions between features. The features were generated using the SMILES of the raw structures tested in vitro, that is, in their salt form. We did not investigate how using SMILES for different forms, i.e., ion, neutral, or salt, would affect the results. However, for some descriptors, especially those influenced by charge, the assumed form can have a strong impact. Interestingly, none of the selected features explicitly included elements related to the sulfonic group or, more generally, sulfur, despite the clear impact of the sulfonic group on PFAS potency, as demonstrated in Fig. 6. This effect is probably masked by the five continuous descriptors that implicitly account for it. On the other hand, the process successfully identified specific ether substructures that are likely critical for a potent PPARα activation (Fig. 5). This highlights the value of combining different types of analysis, in this case QSAR modelling and PCA, when drawing conclusions. Regarding the ECFP fingerprints used, they encode specific substructures as non-unique bits. This creates the possibility of bit collisions, where two different substructures activate the same bit, potentially giving a false positive low BMD. Therefore, when applying the model, congeners with low BMD values should be further examined to assess the chance of false positive activation.

In silico models reflect the patterns present in the available data. To derive more meaningful and generalizable mappings between PFAS structural motifs and PPARα activation, testing across a broader set of congeners covering a wider region of the PFAS chemical space would be required. However, generating a high-quality experimental dataset for a substantially larger number of PFAS congeners would be time-consuming, costly, and not feasible within a reasonable time frame. A key limitation of the present study, from a QSAR modelling perspective, is the relatively small dataset size, which required using all available chemical structures for model development and therefore did not allow the use of an independent test set for validation. To address this, the feature selection process was designed to ensure that the selected features consistently described the observed BMD values in thousands of independent randomized cross-validations. This repeated evaluation reduces the risk of overfitting to a particular split and serves as a quality evaluation tool in the absence of an independent test set. As a result, the final linear model was supported by features that yield consistent performance across diverse training–validation splits. To further increase the reliability of the model, two applicability domain approaches were combined to restrict predictions to inputs that are structurally similar to the panel of 34 PFAS used during model development.

Having established that most of the tested PFAS congeners were able to activate PPARα in vitro, it is important to consider factors that may influence the interpretation of potency estimates. In the context of nominal PFAS concentrations used in the in vitro assays and related cytotoxicity issues, the toxicokinetic variability of PFAS should be considered when interpreting results. When the entire applied concentration can reach the target site and no significant binding to medium components or cellular structures occurs, nominal concentrations can reasonably approximate biologically effective concentrations. However, in most cases, the bioavailable fraction is significantly reduced due to various processes such as degradation, adsorption to plastic surfaces, evaporation, binding to medium constituents, and partitioning into cellular compartments (Groothuis et al. 2015). For PFAS in particular, protein binding in the medium and within the cells has been identified as a major sink for compounds like PFOA, PFBA, PFOS, and PFHxS (Qin et al. 2023). Depending on the experimental system and the lipophilicity of each congener, partitioning into lipids may also contribute to a reduction in the freely available substance. Membrane affinity further complicates dose-response interpretation for intracellular endpoints, as membrane partitioning can reduce intracellular concentrations (Ebert et al. 2020). Interestingly, one of the descriptors selected by the model was TPSA, which is known to be inversely related to membrane permeability. In the model, TPSA has a positive coefficient, meaning that higher TPSA values are associated with increased BMD. This is consistent with the notion that compounds with higher polar surface area have reduced membrane permeability, resulting in lower intracellular concentrations, and thus, reduced potency. Nonetheless, literature examples exist in which high TPSA is observed alongside high lipophilicity and enhanced permeability (Argikar et al. 2022). Additionally, depending on congener structure and transporter expression profiles, PFAS uptake may be influenced by active transport mechanisms including facilitated uptake or efflux. PFAS are recognized substrates for several transporters (Ruggiero et al. 2021; Ryu et al. 2024), and differences in transporter expression across cell lines can influence internalized fractions and thereby affect response measurements. Experimental studies have quantified intracellular PFAS levels and report overall low cellular uptake, with noticeable variability between congeners, suggesting that structural differences drive variations in internalization and partitioning to cellular components (Fragki et al. 2023; Gorrochategui et al. 2014; Rosenmai et al. 2018). Upon cellular entry, PFAS need to reach the nucleus to activate PPARα. This may involve transport via liver fatty acid-binding protein (L-FABP), a cytosolic carrier with reported affinity for PFAS, which can influence nuclear concentrations and ultimately affect receptor activation (Wolfrum et al. 2001; Zhang et al. 2013). Rosenmai et al. describe the lowest cellular concentration for PFBA, and the highest for PFUnA, perfluorododecanoate and perfluorotetradecanoate while incubating HepG2 cells at the same nominal PFAS concentration. For the majority of the tested PFAS and concentrations, the cellular uptake was below 1%. At the same time, PFCA with increasing carbon chain length up to and including PFNA showed a steeper relationship between PPARα activity and cellular concentration than other studied PFAS. This indicates, that the effect of PFAS on PPARα activity is depending not only on the cellular concentration but also on the chemical characteristics of the PFAS (Rosenmai et al. 2018). Taken together, the effect of a PFAS on PPARα activity, determined in a cell-based in vitro assay, seems to be affected by a multitude of parameters and processes.

Following its development, the model was used to screen more than 10,000 PFAS congeners that are included in the PFASSTRUCTV4 list (Williams et al. 2022). Of these, 1,048 congeners were within the AD, and their predicted BMD values for PPARα activation were in a range between 0.3 µM and 420 µM (see Supplementary Table 6). The top ten compounds with the lowest predicted BMD values are listed in Table 2. By assuming PPARα activation being a MIE relevant for PFAS toxicity, the list provided as Supplementary Table 6 could now be a starting point for further research. First, information on potential human exposure would be required for PFAS congeners with the highest predicted PPARα activation potency. This includes data on their industrial applications, production volumes, and relevant exposure routes. If available evidence indicates that relevant human exposure may occur, experimental examination of the toxicological potential of the PFAS congener would be warranted. Several practical prerequisites must also be fulfilled. The PFAS congener must be commercially available for testing, must possess adequate aqueous solubility to allow evaluation in cell-based assays (e.g., to verify the predicted PPARα activation potential), and must have an available analytical standard as well as validated analytical methods for quantification in relevant matrices such as human serum. Only when these conditions are met it is feasible to evaluate the toxicological profile of the PFAS congener in detail.

Before starting extensive toxicity testing in different animal studies, two questions should be experimentally answered first. First, the predicted BMD for PPARα activation should be verified experimentally, e.g., by means of a PPARα transactivation assay. Second, blood serum levels of the PFAS congener of interest should be determined, either in the general population or – if possible – in a highly exposed cohort. Blood serum levels of the PFAS congener of interest should be assessed in relation to blood serum levels of well-characterized, accumulative congeners such as PFOA or PFOS. Due to very low excretion rates, some legacy PFAS (e.g., PFOA and PFOS) display half-times of several years and, thus, tend to accumulate in the human body. As a consequence of continuous exposure to the legacy PFAS for several decades, the accumulative congeners can be detected in human blood serum samples worldwide. In spite of their accumulative potential, blood serum levels of, e.g., PFOA and PFOS are only in the range of 1–10 nM for the general population, and much higher levels in the range of 1 µM are rarely detected usually only in the context of certain contamination incidents (EFSA 2020). If the PFAS congener of interest would be determined in human blood samples in the nM to µM range similar to PFOS or PFOA, this would indicate a relevant exposure, possibly associated with an accumulative potential, posing a concern from the exposure side. If the QSAR model of the present study would on top of this predict a BMD for PPARα activation for this PFAS congener of interest comparable or even lower than that of PFOS or PFOA, this could be assessed as an alert from the hazard perspective. Relevant blood serum levels together with a high PPARα activation potential could then justify a detailed toxicological characterization of this PFAS congener of interest.

HFPO-DA (GenX) can be used as an example to illustrate the proposed strategy for PFAS prioritization in the context of PPARα activation potential. In the present study, the BMD for PPARα activation has been experimentally determined to be 2.38 µM for HFPO-DA compared to 20 µM and 63 µM for PFOA and PFOS, respectively, alerting to a potential hazard of HFPO-DA. With respect to human exposure to HFPO-DA, lessons can be learned from the so-called GenX Exposure Study. In North Carolina, the Cape Fear River, an important drinking water reservoir, has been heavily contaminated with HFPO-DA (GenX) due to the discharge of manufacturing wastewater from a fluorochemical production plant, resulting in significant exposure to HFPO-DA and related PFAS congeners in the surrounding population for several decades. In the GenX Exposure Study, numerous private drinking water wells in the Cape Fear River region were sampled for a number of PFAS, as well as blood serum samples of the owners consuming the water from these wells. The analysis revealed that PFOA and PFOS – that have been no longer used for the production of fluorochemicals for several years – were not detected in the water samples of most of the wells, whereas these two accumulative congeners were still detected in most of the blood serum samples of the well owners (*n* = 153, median 2.7 ng/mL for PFOA and 9.3 ng/mL for PFOS). HFPO-DA, on the other hand, was quantified in 71 of the 84 private wells (median 107 ng/L), but was not detected in any of the 153 blood samples (method reporting limit 0.4–2.5 ng/mL) (Kotlarz et al. 2024). This indicates, that HFPO-DA is not bioaccumulative. Indeed, Abraham et al. have recently determined the half-time for excretion of HFPO-DA in humans to be about 3 days which is in clear contrast to the half-times estimated for, e.g., PFOA (2.3–8.5 years) and PFOS (3.1–7.4 years) (Abraham et al. 2024; EFSA 2020). To the best of our knowledge, HFPO-DA has not been detected in human blood serum samples from cohort studies so far. Thus, the bioavailability data do not support a concern for human health and would not justify a detailed toxicological characterization of HFPO-DA, in spite of HFPO-DA being approximately ten times more potent in PPARα activation than PFOA or PFOS.

Finally, it should be noted that the strategy presented in this study is based on the assumption that PPARα activation is a relevant driver of PFAS toxicity. A positive association between PFAS-mediated PPARα activation and PFAS-induced dysregulation of lipid and cholesterol homoeostasis as well as PFAS-induced adverse effects in rodent liver is well-documented. However, the contribution of PPARα activity on other endpoints, e.g., immune effects or endocrine effects, is less clear. Therefore, the conclusions drawn in the present study may primarily apply to liver-related outcomes. On the other hand, the strategy of the present study may pave the way for additional studies with a focus on other PFAS toxicity endpoints, e.g., related to the immune system. Integrating predictions across multiple models may ultimately provide a more robust basis for identifying PFAS with the highest priority for hazard characterization and finally for risk assessment.

## Conclusions

This study employed a combination of in vitro experiments and in silico modelling to investigate the activation potency of PFAS on PPARα. One of the main findings is that PFAS containing sulfonic groups are less potent in PPARα activation, compared to those containing carboxylic groups. This trend has also been reported in several previous studies. Furthermore, (poly)-ether PFAS belonging to the PFECA subgroup proved to be more potent PPARα agonist than PFCA congeners. Using the in vitro data for subsequent QSAR modeling allowed us to identify a specific substructure (Ether-Carboxyl Bit) within PFECA that appears to drive PPARα activation potency. The QSAR model was used to predict BMD values for about 10,000 PFAS congeners with reliable predictions for about 1,000 of them being within the AD of the model. The congeners with the highest predicted PPARα activation potential may now be prioritized for hazard characterization.

## Supplementary Information

Below is the link to the electronic supplementary material.

Supplementary Material 1

Supplementary Material 2

Supplementary Material 3

## Abbreviations

8:2 Cl-PFESA: 11-chloroeicosafluoro-3-oxaundecane-1-sulfonic acid potassium salt
6:2 FTS: 3,3,4,4,5,5,6,6,7,7,8,8,8-Tridecafluorooctanesulfonic acid
AD: Applicability domain
AOP: Adverse Outcome Pathway
ATSDR: Agency for Toxic Substances and Disease Registry
BMD: Benchmark dose
BMDL: Benchmark dose lower bound
BMDU: Benchmark dose upper bound
BMR: Benchmark response
Branched ADONA: 2,3,3,3-tetrafluoro-2-[1,1,2,3,3,3-hexafluoro-2-(trifluoromethoxy)propoxy]propanoic acid
CH3-PFO3TriDA: Methyl perfluoro-3,6,9-trioxatridecanoate
DLR: Dual luciferase reporter gene
DMEM: Dulbeccos’s modified Eagles’s medium with phenol red
ECFP: Extended connectivity fingerprints
EEA: Ammonium perfluoro-3,6-dioxaoctanoate
EFSA: European Food Safety Authority
FBS: Fetal bovine serum
FLuc: Firefly luciferase
HFPO-TA: 2,3,3,3-Tetrafluoro-2-(1,1,2,3,3,3-hexafluoro-2-(perfluoropropoxy)propoxy)propanoic acid
HFPO-TeA: Perfluoro-2,5,8-trimethyl-3,6,9-trioxadodecanoic acid
IARC: International Agency for Research on Cancer
L-FABP: Liver fatty acid-binding protein
MACCS: Molecular access system
MAE: Mean absolute error
MCMC: Markov Chain Monte Carlo
MIE: Molecular initiating event
MTT: 3-(4,5-dimethylthiazol-2-yl)-2,5-diphenyltetrazolium bromide
Nafion-BP1: Perfluoro-3,6-dioxa-4-methyl-7-octene-1-sulfonic acid
Nafion-BP2: 7H-perfluoro-4-methyl-3,6-dioxaoctanesulfonic acid
OECD: Organisation for Economic Co-operation and Development
PC: Positive control
PCA: Principal component analysis
PF2EOESA: Perfluoro (3-oxapentane-1-sulfonic acid)
PFAS: Per- and polyfluoroalkyl substance
PFBA: Perfluorobutanoic acid
PFBS: Perfluorobutanesulfonic acid
PFCA: Perfluoroalkyl carboxylic acid
PFDA: Perfluorodecanoic acid
PFECA: Perfluoroalkylether carboxylic acid
PFESA: Perfluoroalkylether sulfonic acid
PFHpA: Perfluoroheptanoic acid
PFHpS: Perfluoroheptanesulfonic acid
PFHxA: Perfluorohexanoic acid
PFHxS: Perfluorohexanesulfonic acid potassium salt
PFMOAA: 2,2-difluoro-2-(trifluoromethoxy) acetic acid
PFMOBA: Perfluoro-4-methoxybutanoic acid
PFMOPrA: Perfluoro-3-methoxypropanoic acid
PFNA: Perfluorononanoic acid
PFO2DA: Perfluoro-3,6-dioxadecanoic acid
PFO3DA: Perfluoro-3,6,9-trioxadecanoic acid
PFO3TriDA: Perfluoro-3,6,9-trioxatridecanoic acid
PFOA: Perfluorooctanoic acid
PFOS: Perfluorooctanesulfonic acid
PFoxaOA: Ammonium 2-perfluoropentoxy-2,3,3,3-tetrafluoropropanoate
PFPeA: Perfluoropentanoic acid
PFPeS: Perfluoropentanesulfonic acid
PFPrA: Pentafluoropropionic acid
PFSA: Perfluoroalkyl sulfonic acid
PFUnA: Perfluoroundecanoic acid
PPARα: Peroxisome proliferator-activated receptor alpha
QMRF: QSAR model reporting format
R^2^: Coefficient of determination
RLuc: *Renilla* luciferase
RMSE: Root mean squared error
SC: Solvent control
Similar to Nafion-BP2: Potassium perfluoro(4-methyl-3,6-dioxaoctane)sulfonate
TG: Test Guideline
TPSA: Topological polar surface area
