# Supplementary material for: Development of a QSAR model for predicting PPARα activation by PFAS based on human in vitro data of a comprehensive panel of legacy and novel PFAS
Source: Arch Toxicol. 2026 Mar 31;100(5):2047–67. doi: 10.1007/s00204-026-04359-2 (PMC13086658; doi:10.1007/s00204-026-04359-2)
Supplement: Supplementary file 1 — Supplementary Material 1 [file 204_2026_4359_MOESM1_ESM.pdf]

Development of a QSAR model for predicting PPARα activation by PFAS based on human *in vitro* data of a comprehensive panel of legacy and novel PFAS

Wiebke Alker[1*], Periklis Tsiros[2*], Haralambos Sarimveis[2], Albert Braeuning[1], Thorsten Buhrke[1#]

[1] German Federal Institute for Risk Assessment (BfR), Department Chemical and Product Safety, Max-Dohrn-Str. 8-10, 10589 Berlin, Germany
[2] National Technical University of Athens, School of Chemical Engineering, 9 Iroon Polytechniou Str, 15772, Athens, Greece
[*] These authors contributed equally to this work.

[#] Corresponding author:
Dr. Thorsten Buhrke, German Federal Institute for Risk Assessment (BfR), Department Chemical and Product Safety, Max-Dohrn-Str. 8-10, 10589 Berlin, Germany, e-mail thorsten.buhrke@bfr.bund.de

Supplementary Figures 1 – 2

Supplementary Figure 1 Cellular viability relative to untreated solvent control of HEK293T cells, determined by MTT-assay after 24h of incubation with PFAS congeners (A: PFCA, B: PFSA; C: PFECA, D: PFESA and fluorotelomer sulfonic acid) and concentrations as indicated. Data are shown as mean + standard deviation of three biological replicates with each three technical replicates

A

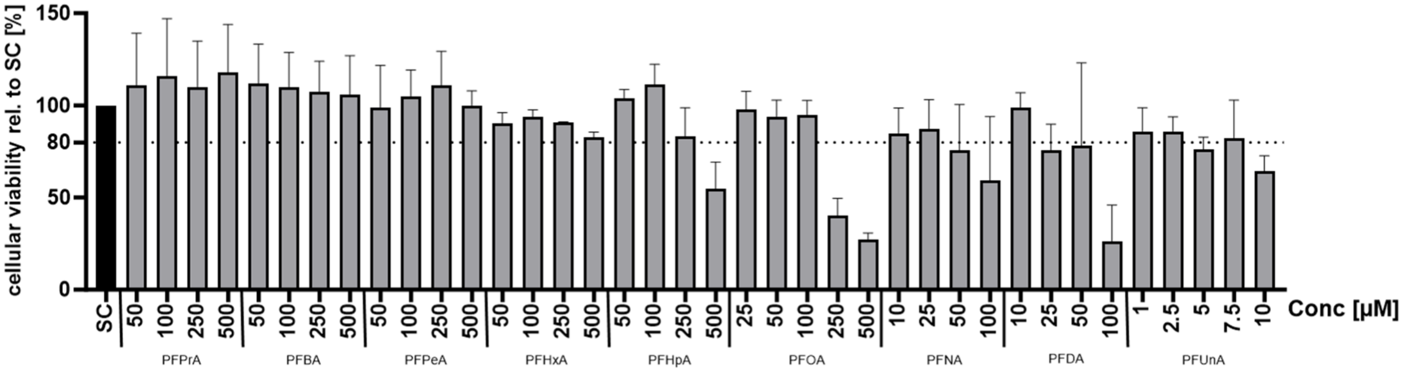

B

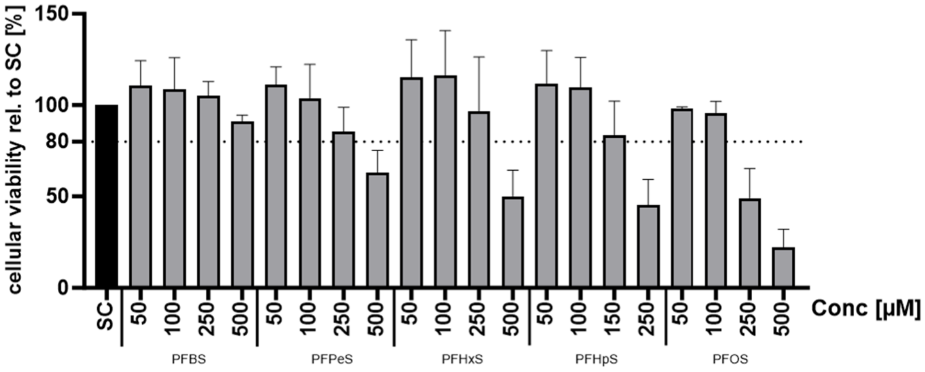

C

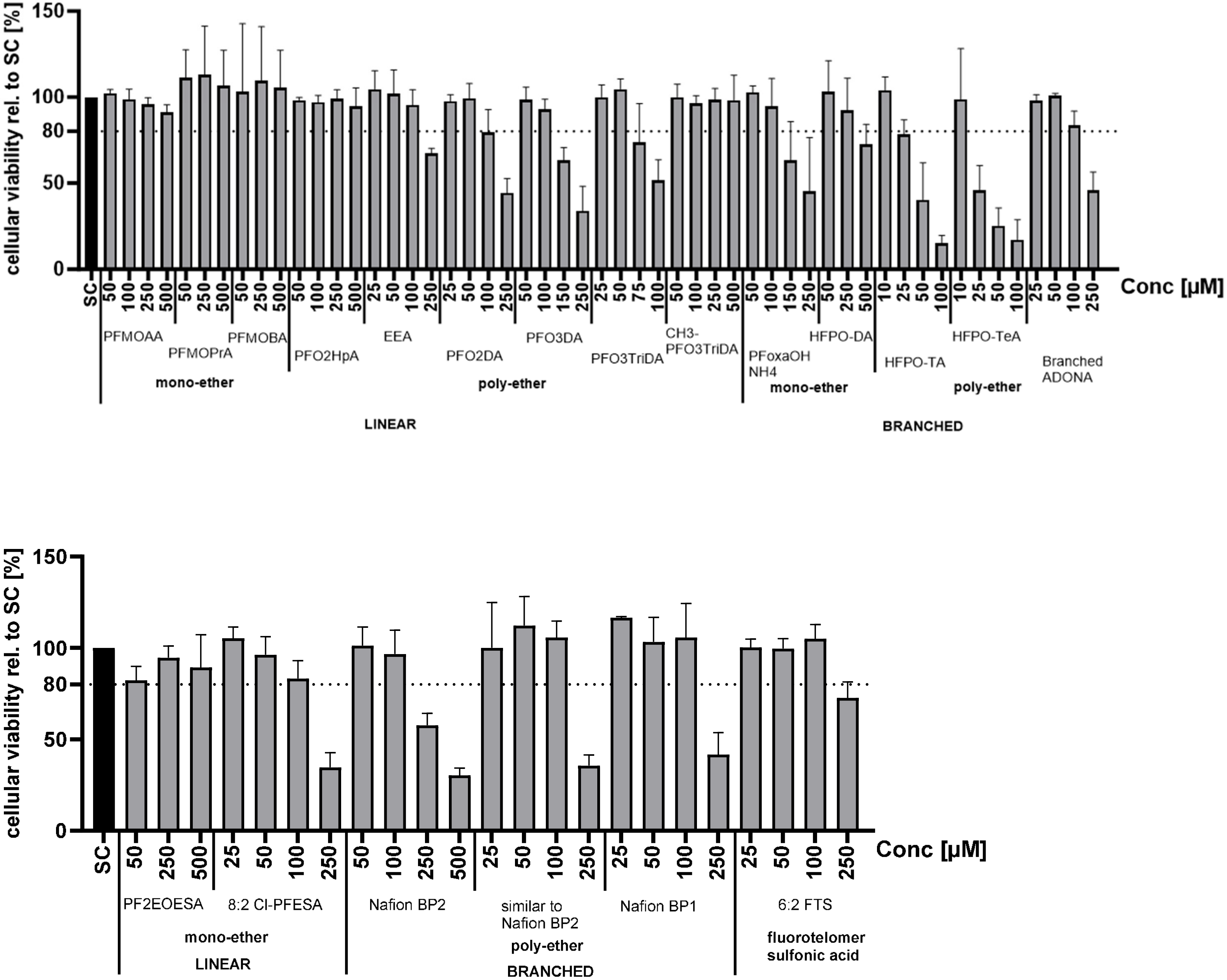
cellular viability rel. to SC [%]
150
100
80
50
0
Conc [µM]
SC
PFMOAA
PFMOPrA
PFMOBA
PFO2HpA
EEA
PFO2DA
PFO3DA
PFO3TriDA
CH3-PFO3TriDA
PFoxaOH NH4
HFPO-DA
HFPO-TA
HFPO-TeA
Branched ADONA
mono-ether
poly-ether
LINEAR
mono-ether
poly-ether
BRANCHED
cellular viability rel. to SC [%]
150
100
80
50
0
Conc [µM]
SC
PF2EOESA
8:2 Cl-PFESA
Nafion BP2
similar to Nafion BP2
Nafion BP1
6:2 FTS
mono-ether
LINEAR
poly-ether
BRANCHED
fluorotelomer sulfonic acid

Supplementary Figure 2 HEK293T cells were co-transfected with plasmids pGAL4-(UAS)5-TK-LUC, pGAL4-hPPARα-LBD and pcDNA3-Rluc for 5h. Cells were then incubated with PFAS congeners (A1, A2: PFCA, B: PFSA; C1, C2: PFECA, D1, D2: PFESA and fluorotelomer sulfonic acid) and concentrations for 24h as indicated before luciferase activity was measured. Values were normalized to *Renilla* luciferase activity and compared to the value of the untreated solvent control. Data are shown as mean + standard deviation of three biological replicates with each three technical replicates. Dotted line shows benchmark response (BMR) of 1.5

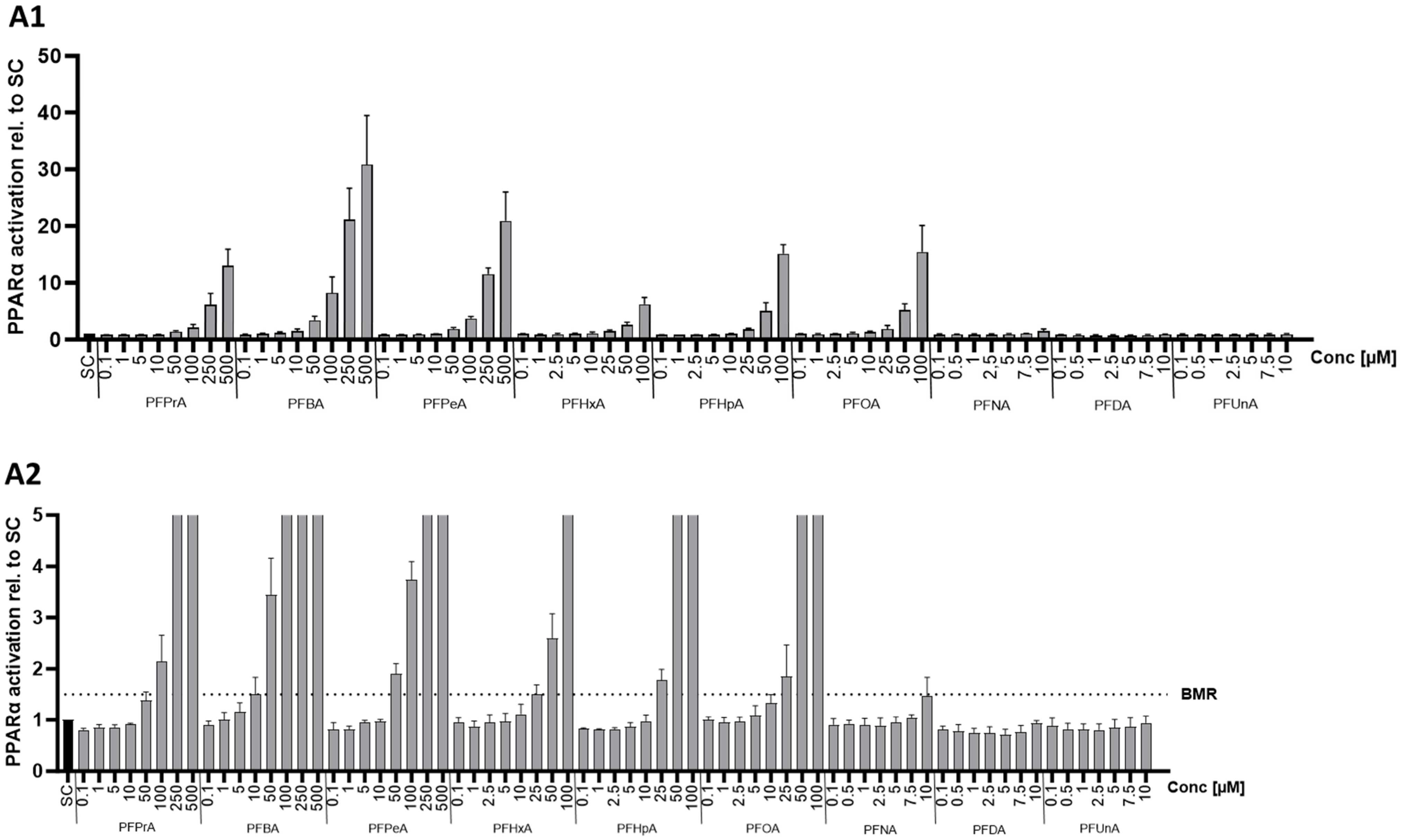

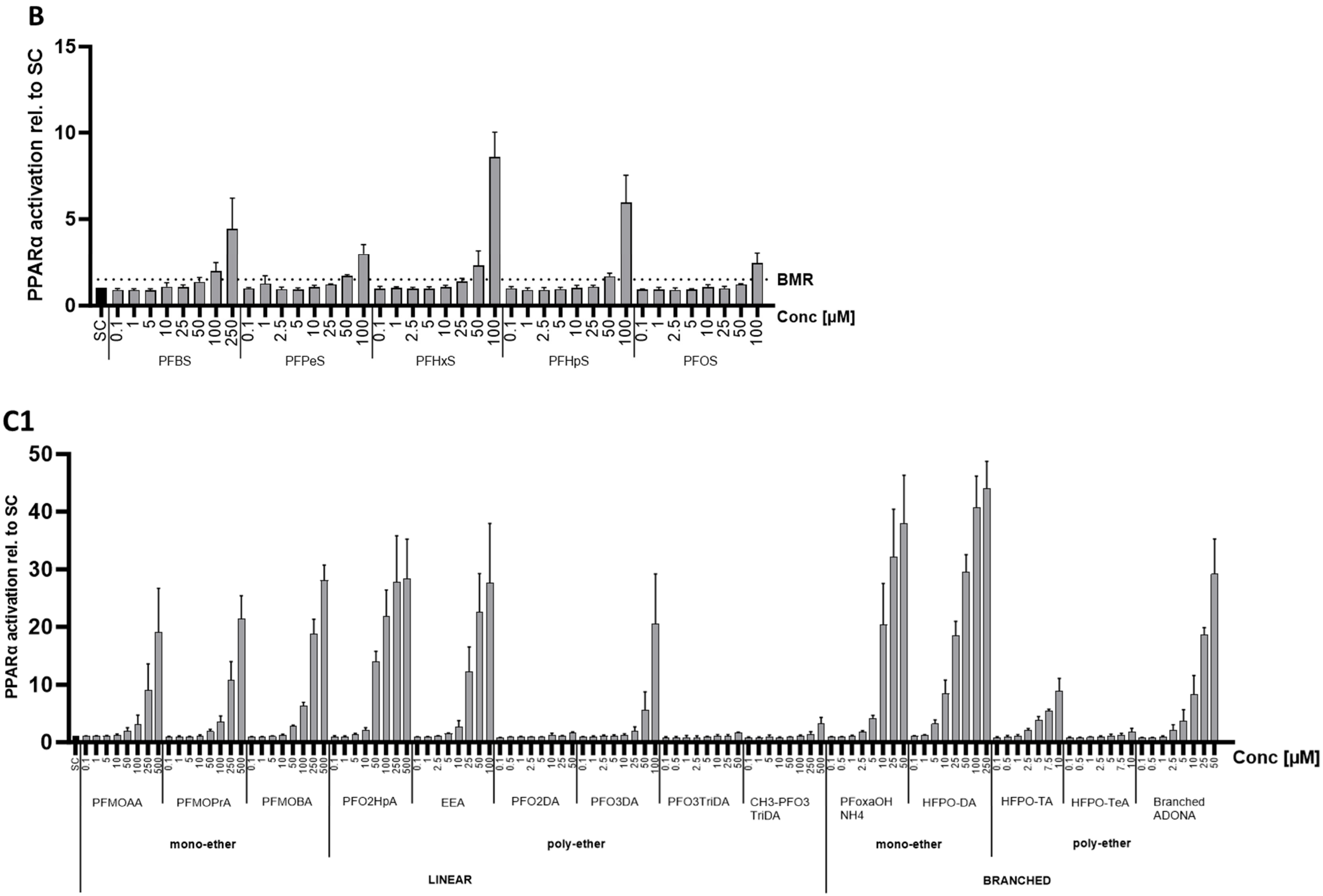

B
PPARα activation rel. to SC
15
10
5
0
BMR
Conc [µM]
SC
PFBS
PFPeS
PFHxS
PFHpS
PFOS
C1
PPARα activation rel. to SC
50
40
30
20
10
0
Conc [µM]
PFMOAA
PFMOPrA
PFMOBA
PFO2HpA
EEA
PFO2DA
PFO3DA
PFO3TriDA
CH3-PFO3 TriDA
PFoxaOH NH4
HFPO-DA
HFPO-TA
HFPO-TeA
Branched ADONA
mono-ether
poly-ether
mono-ether
poly-ether
LINEAR
BRANCHED

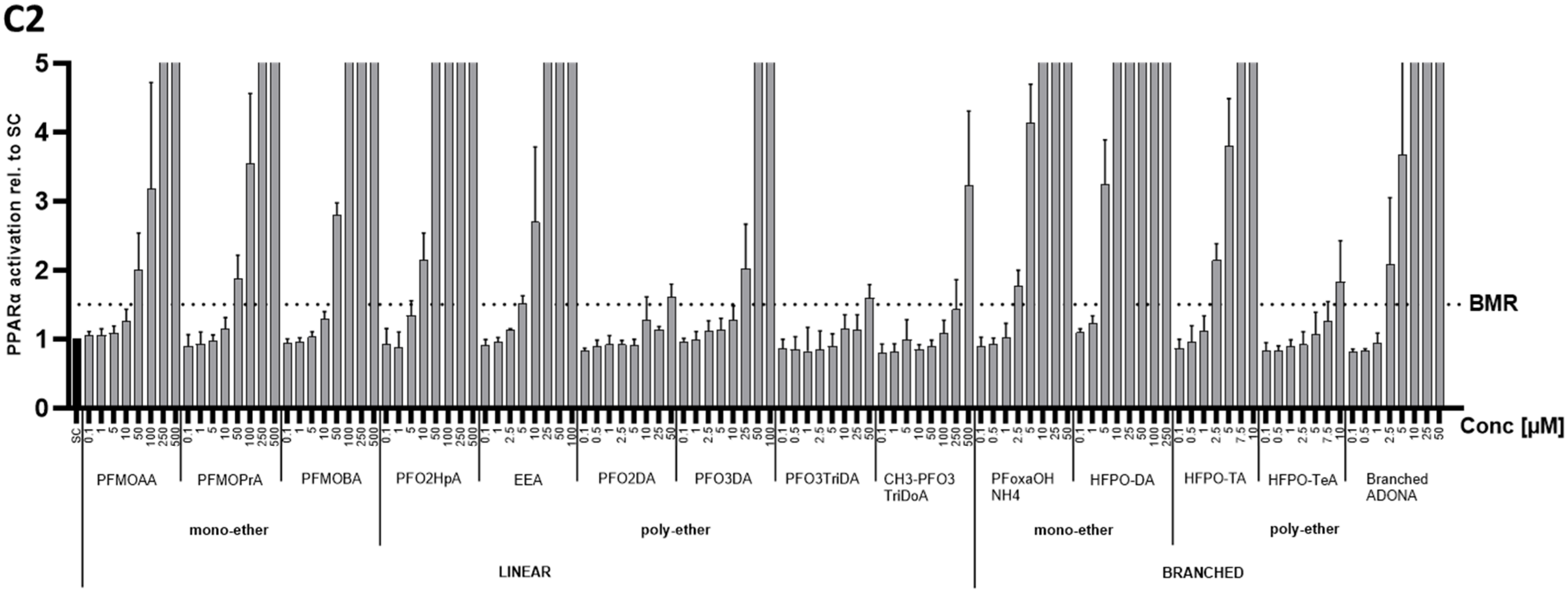
C2
PPARα activation rel. to SC
BMR
Conc [µM]
SC
PFMOAA
PFMOPrA
PFMOBA
PFO2HpA
EEA
PFO2DA
PFO3DA
PFO3TriDA
CH3-PFO3 TriDoA
PFoxaOH NH4
HFPO-DA
HFPO-TA
HFPO-TeA
Branched ADONA
mono-ether
poly-ether
mono-ether
poly-ether
LINEAR
BRANCHED

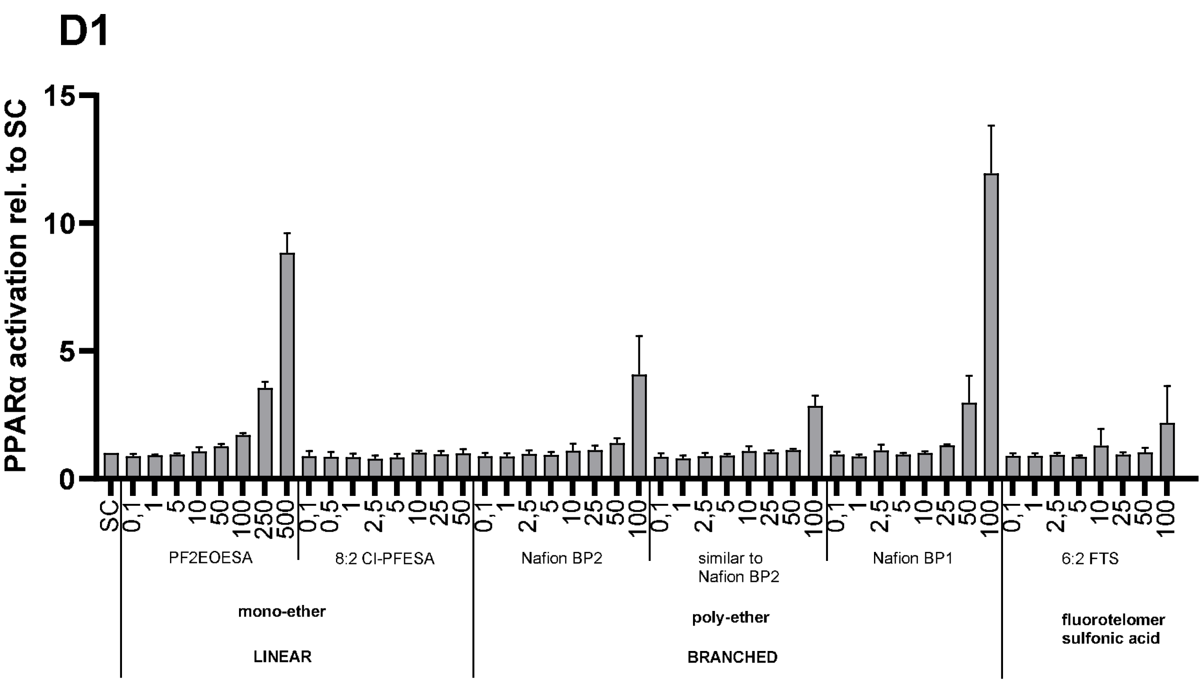
D1
PPARα activation rel. to SC
15
10
5
0
SC
PF2EOESA
8:2 Cl-PFESA
Nafion BP2
similar to Nafion BP2
Nafion BP1
6:2 FTS
mono-ether
poly-ether
fluorotelomer sulfonic acid
LINEAR
BRANCHED

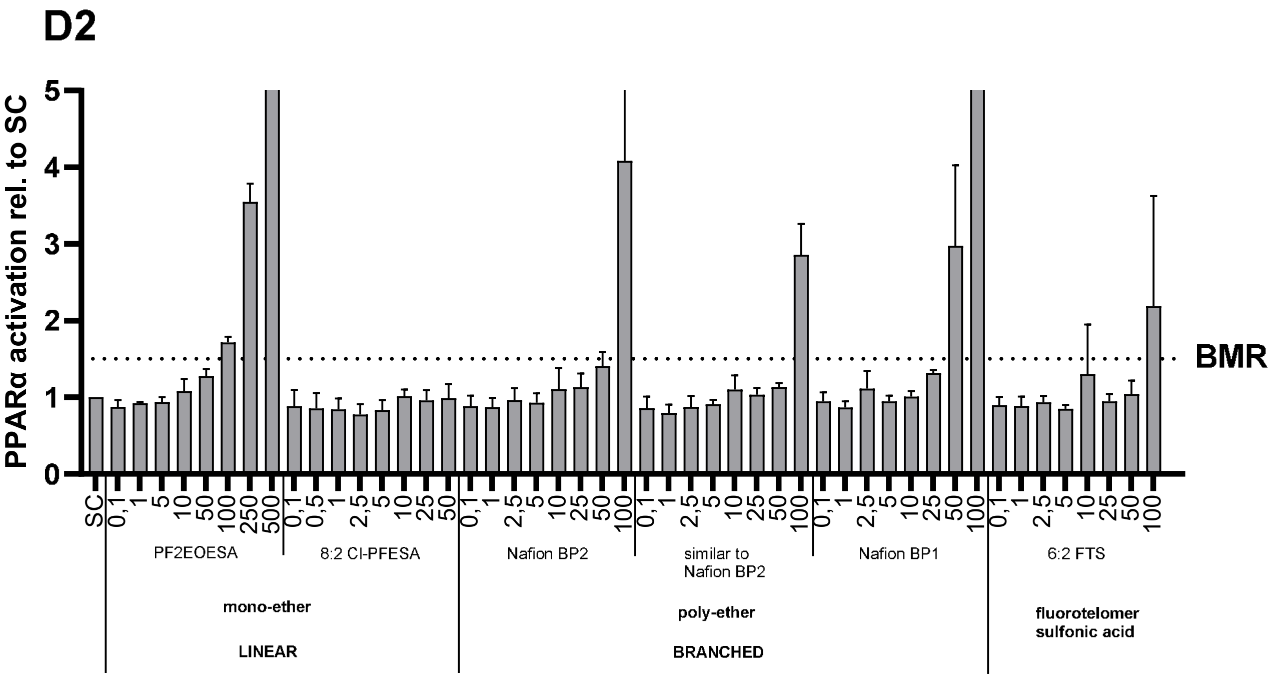
D2
PPARα activation rel. to SC
5
4
3
2
1
0
BMR
SC
PF2EOESA
8:2 Cl-PFESA
Nafion BP2
similar to Nafion BP2
Nafion BP1
6:2 FTS
mono-ether
poly-ether
fluorotelomer sulfonic acid
LINEAR
BRANCHED
