# Supplementary material for: Development of a QSAR model for predicting PPARα activation by PFAS based on human in vitro data of a comprehensive panel of legacy and novel PFAS
Source: Arch Toxicol. 2026 Mar 31;100(5):2047–67. doi: 10.1007/s00204-026-04359-2 (PMC13086658; doi:10.1007/s00204-026-04359-2)
Supplement: Supplementary file 2 — Supplementary Material 2 [file 204_2026_4359_MOESM2_ESM.pdf]

Development of a QSAR model for predicting PPARα activation by PFAS based on human *in vitro* data of a comprehensive panel of legacy and novel PFAS

Wiebke Alker[1*], Periklis Tsiros[2*], Haralambos Sarimveis[2], Albert Braeuning[1], Thorsten Buhrke[1#]

[1] German Federal Institute for Risk Assessment (BfR), Department Chemical and Product Safety, Max-Dohrn-Str. 8-10, 10589 Berlin, Germany

[2] National Technical University of Athens, School of Chemical Engineering, 9 Iroon Polytechniou Str, 15772, Athens, Greece

[*] These authors contributed equally to this work.

[#] Corresponding author:

Dr. Thorsten Buhrke, German Federal Institute for Risk Assessment (BfR), Department Chemical and Product Safety, Max-Dohrn-Str. 8-10, 10589 Berlin, Germany, e-mail thorsten.buhrke@bfr.bund.de

Supplementary Tables 1 – 5

Supplementary Table 1 Structure and CAS number of the 34 PFAS congeners used in this study.

PFAS Subgroup	Name	Abbreviation	Structure	CAS no.
PFCA	Perfluoropropionic acid	PFPrA	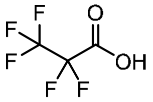	422-64-0
PFCA	Perfluorobutanoic acid	PFBA	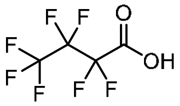	375-22-4
PFCA	Perfluoropentanoic acid	PFPeA	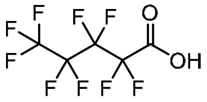	2706-90-3
PFCA	Perfluorohexanoic acid	PFHxA	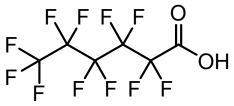	307-24-4
PFCA	Perfluoroheptanoic acid	PFHpA	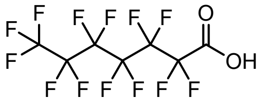	375-85-9
PFCA	Perfluorooctanoic acid	PFOA	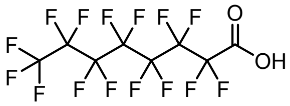	335-67-1
PFCA	Perfluorononanoic acid	PFNA	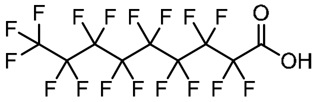	375-95-1
PFCA	Perfluorodecanoic acid	PFDA	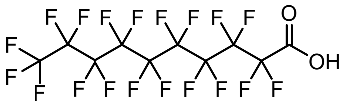	335-76-2
PFCA	Perfluoroundecanoic acid	PFUnA	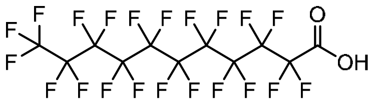	2058-94-8
PFSA	Perfluorobutanesulfonic acid	PFBS	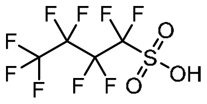	375-73-5
PFSA	Perfluoropentanesulfonic acid	PFPeS	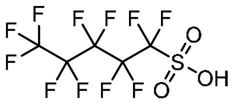	2706-91-4
PFSA	Perfluorohexanesulfonic acid potassium salt	PFHxS	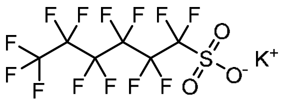	3871-99-6

PFSA	Perfluoroheptanesulfonic acid	PFHpS	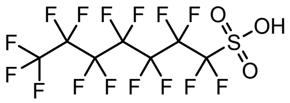	375-92-8
PFSA	Perfluorooctanesulfonic acid potassium salt	PFOS	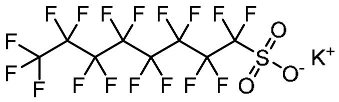	2795-39-3
PFECA linear monoether	2,2-difluoro-2-(trifluoromethoxy) acetic acid	PFMOAA	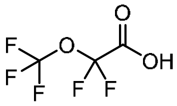	674-13-5
PFECA linear monoether	Perfluoro-3-methoxypropanoic acid	PFMOPrA	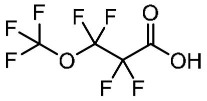	377-73-1
PFECA linear monoether	Perfluoro-4-methoxybutanoic acid	PFMOBA	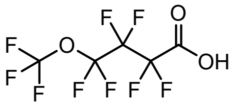	863090-89-5
PFECA linear polyether	Perfluoro-3,6-dioxaheptanoic acid	PFO2HpA	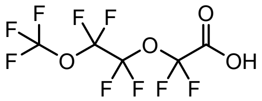	151772-58-6
PFECA linear polyether	Ammonium perfluoro-3,6-dioxaoctanoate	EEA	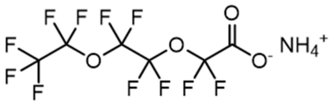	908020-52-0
PFECA linear polyether	Perfluoro-3,6-dioxadecanoic acid	PFO2DA	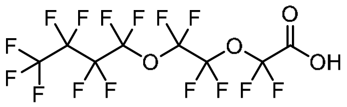	137780-69-9
PFECA linear polyether	Perfluoro-3,6,9-trioxadecanoic acid	PFO3DA	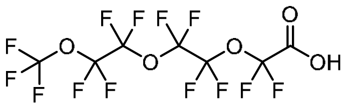	151772-59-7
PFECA linear polyether	Perfluoro-3,6,9-trioxatridecanoic acid	PFO3TriDA	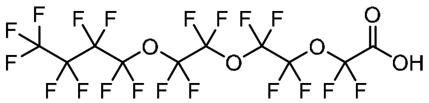	330562-41-9
PFECA linear polyether	Methyl perfluoro-3,6,9-trioxatridecanoate	CH3-PFO3TriDA	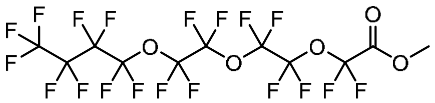	330562-42-0
PFECA branched monoether	2,3,3,3-Tetrafluoro-2-(heptafluoropropoxy)-propanoic acid	HFPO-DA	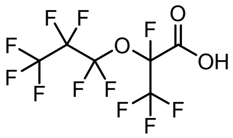	13252-13-6

PFECA branched monoether	Ammonium 2-perfluoropentoxy-2,3,3,3-tetrafluoropropanoate	PFoxaOA	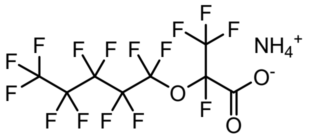	96513-97-2
PFECA branched polyether	Perfluoro-2,5-dimethyl-3,6-dioxaheptanoic acid	Branched ADONA	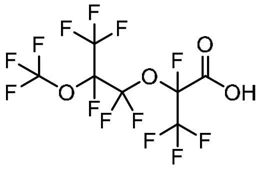	2479-73-4
PFECA branched polyether	Perfluoro-2,5-dimethyl-3,6-dioxaheptanoic acid	HFPO-TA	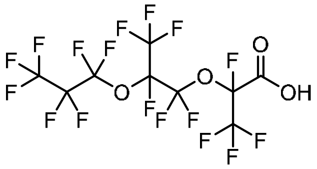	13252-14-7
PFECA branched polyether	Perfluoro-2,5,8-trimethyl-3,6,9-trioxadodecanoic acid	HFPO-TeA	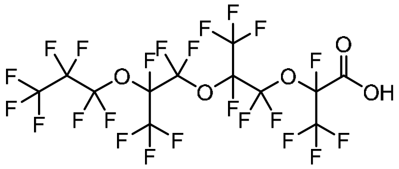	65294-16-8
PFESA linear monoether	Perfluoro-3-oxapentane-1-sulfonic acid	PF2EOESA	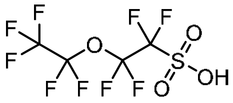	113507-82-7
PFESA linear monoether	11-chloroeicosafluoro-3-oxaundecane-1-sulfonic acid	8:2 Cl-PFESA	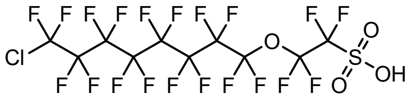	763051-92-9
PFESA branched polyether	7H-Perfluoro-4-methyl-3,6-dioxaoctanesulfonic acid	Nafion-BP2	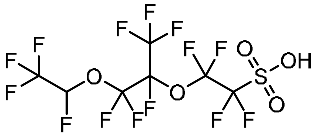	749836-20-2
PFESA branched polyether	Potassium perfluoro(4-methyl-3,6-dioxaoctane)-sulfonate	similar to Nafion-BP2	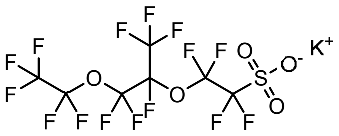	70755-50-9
PFESA branched polyether	Perfluoro-3,6-dioxa-4-methyl-7-octene-1-sulfonic acid	Nafion-BP1	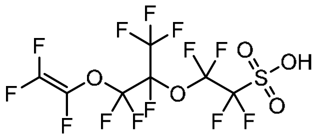	29311-67-9
Fluorotelomer sulfonic acid	1H,1H,2H,2H-Perfluorooctanesulfonic acid	6:2 FTS	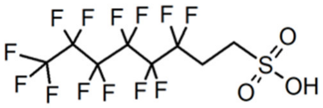	27619-97-2

Supplementary Table 2 Benchmark dose (BMD), lower confidence bound (BMDL), and upper confidence bound (BMDU) estimates for the 34 PFAS included in the study. The BMDU/BMDL ratio is also reported as an indicator of uncertainty associated with each estimate. A dash (-) indicates that no BMD estimate could be derived due to the absence of a clear ascending dose-response relationship.

PFAS Subgroup	Congener	PubChem CID	BMD	BMDL	BMDU	BMDU/BMDL
PFCA	PFPrA	62356	68.65	59.19	78.83	1.33
PFCA	PFBA	9777	15.16	11.89	19.06	1.60
PFCA	PFPeA	75921	37.25	32.72	41.80	1.28
PFCA	PFHxA	67542	28.97	25.86	32.59	1.26
PFCA	PFHpA	67818	23.65	21.25	26.25	1.24
PFCA	PFOA	9554	20.33	17.67	23.34	1.32
PFCA	PFNA	67821	10.58	9.75	14.10	1.45
PFCA	PFDA	9555	-	-	-	-
PFCA	PFUnA	77222	-	-	-	-
PFSA	PFBS	67815	69.24	60.89	79.48	1.31
PFSA	PFPeS	75922	39.32	34.87	45.54	1.31
PFSA	PFHxS	23678874	34.74	31.53	37.74	1.20
PFSA	PFHpS	67820	46.54	43.79	49.45	1.13
PFSA	PFOS	23669238	63.14	57.51	68.43	1.19
PFECA linear monoether	PFMOAA	5125273	36.13	28.38	49.21	1.73
PFECA linear monoether	PFMOPrA	120228	36.02	30.87	41.26	1.34

PFECA linear monoether	PFMOBA	12498036	20.24	18.29	22.41	1.23
PFECA linear polyether	PFO2HpA	2782393	7.21	6.38	8.00	1.25
PFECA linear polyether	EEA	46221768	6.11	5.28	6.94	1.31
PFECA linear polyether	PFO2DA	2778677	52.09	33.07	3515.52	106.31
PFECA linear polyether	PFO3DA	2778260	19.58	15.79	23.49	1.49
PFECA linear polyether	PFO3TriDA	2760333	42.95	34.75	50.88	1.46
PFECA linear polyether	CH3-PFO3TriDA	2769352	276.00	242.32	310.81	1.28
PFECA branched monoether	PFoxaOA	2782557	2.45	2.18	2.78	1.28
PFECA branched monoether	HFPO-DA (GenX)	114481	2.38	2.10	2.72	1.30
PFECA branched polyether	HFPO-TA	93076	1.86	1.66	2.11	1.27
PFECA branched polyether	HFPO-TeA	182328	9.26	8.43	10.68	1.27
PFECA branched polyether	Branched ADONA	87119578	2.16	1.97	2.52	1.28

PFESA linear monoether	PF2EOESA	277618	84.82	76.01	94.96	1.25
PFESA linear monoether	8:2 Cl-PFESA	50851128	-	-	-	-
PFESA branched polyether	Nafion-BP2	14317645	53.12	49.04	58.50	1.19
PFESA branched polyether	similar to Nafion-BP2	23671509	63.36	57.21	72.72	1.27
PFESA branched polyether	Nafion BP1	61890	33.28	29.94	36.68	1.23
Fluorotelomer sulfonic acid	6:2 FTS	119688	-	-	-	-

Supplementary Table 3 Overview of the final PFAS congeners used for QSAR modelling, including their SMILES representations, the corresponding design matrix comprising the seven selected computational descriptors, and the associated BMD values. Within the subgroup of PFCA and PFSA the congeners are sorted by increasing chain length. This allows to understand the effect of this structural feature on the selected descriptors. For example, a comparison of PFOA and PFOS shows how of a carboxylic or sulfonic group as a functional group effects the selected descriptors, as both congeners have a carbon chain length equal to eight.

PFAS Subgroup	Congener	SMILES	TPSA	ATSC6c	ATSC5dv	AATS5dv	GATS5p	Ether-Carboxyl Bit	Ether Bit	BMD
PFCA	PFPrA	OC(=O)C(F)(F)C(F)(F)F	37.3	0	-27.32	0	0.02	0	0	68.6
PFCA	PFBA	OC(=O)C(F)(F)C(F)(F)C(F)(F)F	37.3	-0.15	-8.63	25.67	0.31	0	0	15.2
PFCA	PFPeA	OC(=O)C(F)(F)C(F)(F)C(F)(F)C(F)(F)F	37.3	0.38	-1.66	32.00	0.71	0	0	37.2
PFCA	PFHxA	OC(=O)C(F)(F)C(F)(F)C(F)(F)C(F)(F)C(F)(F)F	37.3	-0.06	-0.48	33.33	0.82	0	0	29.0
PFCA	PFHpA	OC(=O)C(F)(F)C(F)(F)C(F)(F)C(F)(F)C(F)(F)C(F)(F)F	37.3	-0.03	0.18	34.00	0.87	0	0	23.7
PFCA	PFOA	OC(=O)C(F)(F)C(F)(F)C(F)(F)C(F)(F)C(F)(F)C(F)(F)C(F)(F)F	37.3	-0.002	0.57	34.40	0.90	0	0	20.3
PFCA	PFNA	OC(=O)C(F)(F)C(F)(F)C(F)(F)C(F)(F)C(F)(F)C(F)(F)C(F)(F)C(F)(F)F	37.3	0.0008	0.80	34.67	0.92	0	0	10.6

PFSA	PFBS	OS(=O)(=O)C(F)(F)C(F)(F)C(F)(F)C(F)(F)F	54.4	0.35	-15.53	29.24	1.10	0	0	69.2
PFSA	PFPeS	OS(=O)(=O)C(F)(F)C(F)(F)C(F)(F)C(F)(F)C(F)(F)F	54.4	-0.04	-3.14	31.60	0.90	0	0	39.3
PFSA	PFHxS	[K+].[O-]S(=O)(=O)C(F)(F)C(F)(F)C(F)(F)C(F)(F)C(F)(F)C(F)(F)F	57.2	-0.06	6.22	36.33	0.01	0	0	34.7
PFSA	PFHpS	OS(=O)(=O)C(F)(F)C(F)(F)C(F)(F)C(F)(F)C(F)(F)C(F)(F)C(F)(F)F	54.4	-0.003	-0.78	33.25	0.88	0	0	46.5
PFSA	PFOS	[K+].[O-]S(=O)(=O)C(F)(F)C(F)(F)C(F)(F)C(F)(F)C(F)(F)C(F)(F)C(F)(F)C(F)(F)F	57.2	-0.0001	5.85	36.22	0.01	0	0	63.1
PFECA linear monoether	PFMOAA	OC(=O)C(F)(F)OC(F)(F)F	46.5	-0.13	8.78	33.00	0.44	0	0	36.1
PFECA linear monoether	PFMOPrA	OC(=O)C(F)(F)C(F)(F)OC(F)(F)F	46.5	0.47	-12.42	30.14	0.80	0	0	36.0
PFECA linear monoether	PFMOBA	OC(=O)C(F)(F)C(F)(F)C(F)(F)OC(F)(F)F	46.5	-0.55	-4.07	31.90	0.74	0	0	20.2

PFECA linear polyether	PFO2HpA	OC(=O)C(F)(F)OC(F)(F)C(F)(F)OC(F)(F)F	55.8	0.27	29.14	38.23	0.53	0	0	7.2
PFECA linear polyether	EEA	C(=O)(C(OC(C(OC(C(F)(F)F)(F)F)(F)F)(F)F)(F)F)[O-].[NH4+]	95.1	0.87	72.34	39.65	0.87	0	0	6.1
PFECA linear polyether	PFO2DA	OC(=O)C(F)(F)OC(F)(F)C(F)(F)OC(F)(F)C(F)(F)C(F)(F)C(F)(F)F	55.8	0.28	27.55	37.23	0.74	0	1	52.1
PFECA linear polyether	PFO3DA	OC(=O)C(F)(F)OC(F)(F)C(F)(F)OC(F)(F)C(F)(F)OC(F)(F)F	65.0	0.72	34.57	38.44	0.62	0	0	19.6
PFECA linear polyether	PFO3TriDA	OC(=O)C(F)(F)OC(F)(F)C(F)(F)OC(F)(F)C(F)(F)OC(F)(F)C(F)(F)C(F)(F)C(F)(F)F	65.0	0.72	32.65	37.67	0.74	0	1	42.9
PFECA linear polyether	CH3-PFO3TriDA	COC(=O)C(F)(F)OC(F)(F)C(F)(F)OC(F)(F)C(F)(F)OC(F)(F)C(F)(F)C(F)(F)C(F)(F)F	54.0	0.78	-1.03	33.44	0.63	0	1	276.0
PFECA branched monoether	PFoxaOA	[NH4+].[O-]C(=O)C(F)(OC(F)(F)C(F)(F)C(F)(F)C(F)(F)C(F)(F)F)C(F)(F)F	85.9	0.32	53.76	37.60	1.04	1	1	2.4
PFECA branched monoether	HFPO-DA (GenX)	OC(=O)C(F)(OC(F)(F)C(F)(F)C(F)(F)F)C(F)(F)F	46.5	-0.40	-18.55	30.00	1.07	1	0	2.4

PFECA branched polyether	HFPO-TA	OC(=O)C(F)(OC(F)(F)C(F)(OC(F)(F)C(F)(F)C(F)(F)F)C(F)(F)F)C(F)(F)F	55.8	-0.43	-15.39	32.63	1.06	1	0	1.9
PFECA branched polyether	HFPO-TeA	OC(=O)C(F)(OC(F)(F)C(F)(OC(F)(F)C(F)(OC(F)(F)C(F)(F)C(F)(F)F)C(F)(F)F)C(F)(F)F)C(F)(F)F	65.0	-0.46	-13.68	33.51	1.05	1	0	9.3
PFECA branched polyether	Branched ADONA	OC(=O)C(F)(OC(F)(F)C(F)(OC(F)(F)F)C(F)(F)F)C(F)(F)F	55.8	-0.17	14.16	35.84	0.65	1	0	2.2
PFESA linear monoether	PF2EOESA	OS(=O)(=O)C(F)(F)C(F)(F)OC(F)(F)C(F)(F)F	63.6	0.01	-15.78	29.58	1.06	0	0	84.8
PFESA branched polyether	Nafion-BP2	[H]C(F)(OC(F)(F)C(F)(OC(F)(F)C(F)(F)S(O)(=O)=O)C(F)(F)F)C(F)(F)F	72.8	-0.34	-6.89	30.29	0.89	0	0	53.1
PFESA branched polyether	similar to Nafion-BP2	[K+].[O-]S(=O)(=O)C(F)(F)C(F)(F)OC(F)(C(F)(F)F)C(F)(F)OC(F)(F)C(F)(F)F	75.7	-0.61	8.17	34.96	0.01	0	0	63.4
PFESA branched polyether	Nafion BP1	OS(=O)(=O)C(F)(F)C(F)(F)OC(F)(C(F)(F)F)C(F)(F)OC(F)=C(F)F	72.8	-0.35	-5.35	31.94	0.89	0	0	33.3

Supplementary Table 4 QSAR-predicted versus observed BMD values obtained during 5-fold cross-validation. For each compound, predictions were generated in a fold where the compound was excluded from the training data, thereby providing an assessment of the model's predictive ability. The table also includes applicability domain evaluations using the bounding box and leverage methods, each reported as TRUE (within domain) or FALSE (outside domain). The applicability domains were computed based on the seven selected descriptors, rather than the full initial descriptor set. The total applicability domain represents the intersection of the two criteria, indicating whether a prediction is considered reliable based on both AD definitions.

**PFAS Subgroup**	**PFAS Congener**	**Predictions [µM]**	**Observations [µM]**	**Bounding Box**	**Leverage**	**Total Applicability Domain**
PFCA	PFPrA	4.3	68.7	FALSE	FALSE	FALSE
PFCA	PFBA	33.0	15.2	TRUE	TRUE	TRUE
PFCA	PFPeA	23.9	37.3	TRUE	TRUE	TRUE
PFCA	PFHxA	17.6	29.0	TRUE	TRUE	TRUE
PFCA	PFHpA	17.4	23.7	TRUE	TRUE	TRUE
PFCA	PFOA	16.5	20.3	TRUE	TRUE	TRUE
PFCA	PFNA	16.9	10.6	TRUE	TRUE	TRUE
PFCA	PFBS	51.5	69.2	FALSE	TRUE	FALSE
PFSA	PFPeS	31.4	39.3	TRUE	TRUE	TRUE
PFSA	PFHxS	62.5	34.7	FALSE	TRUE	FALSE
PFSA	PFHpS	30.5	46.5	TRUE	TRUE	TRUE
PFSA	PFOS	51.9	63.1	TRUE	TRUE	TRUE
PFECA linear monoether	PFMOAA	18.6	36.1	TRUE	TRUE	TRUE
PFECA linear monoether	PFMOPrA	64.4	36.0	TRUE	TRUE	TRUE

PFECA linear monoether	PFMOBA	23.8	20.2	TRUE	TRUE	TRUE
PFECA linear polyether	PFO2HpA	13.4	7.2	TRUE	TRUE	TRUE
PFECA linear polyether	EEA	2.8	6.1	FALSE	FALSE	FALSE
PFECA linear polyether	PFO2DA	37.0	52.1	TRUE	TRUE	TRUE
PFECA linear polyether	PFO3DA	11.0	19.6	FALSE	FALSE	FALSE
PFECA linear polyether	PFO3TriDA	76.9	43.0	TRUE	TRUE	TRUE
PFECA linear polyether	CH3-PFO3TriDA	265.5	276.0	FALSE	FALSE	FALSE
PFECA branched monoether	PFoxaOA	2.0	2.5	TRUE	FALSE	FALSE
PFECA branched monoether	HFPO-DA (GenX)	5.5	2.4	TRUE	TRUE	TRUE
PFECA branched polyether	HFPO-TA	6.7	1.9	TRUE	TRUE	TRUE
PFECA branched polyether	HFPO-TeA	4.2	9.3	TRUE	TRUE	TRUE
PFECA branched polyether	Branched ADONA	2.6	2.2	TRUE	TRUE	TRUE

PFESA linear monoether	PF2EOESA	56.1	84.8	TRUE	TRUE	TRUE
PFESA branched polyether	Nafion-BP2	45.1	53.1	TRUE	TRUE	TRUE
PFESA branched polyether	similar to Nafion-BP2	42.4	63.4	FALSE	TRUE	FALSE
PFESA branched polyether	Nafion-BP1	50.0	33.3	TRUE	TRUE	TRUE
Fluorotelomer sulfonic acid	6:2 FTS	13.0	4.2	TRUE	TRUE	TRUE

Supplementary Table 5 QSAR model reporting format (QMRF) report for the PPARα activation QSAR model.

	Element	Explanation
**1.**	**QSAR identifier**	
1.1.	QSAR identifier (title)	QSAR model for PPARα activation by PFAS.
1.2	Other related models	-NA-
1.3.	Software coding the model	Python, rdkit, Mordred
**2.**	**General information**	
2.0	Abstract	A simple linear model was developed for predicting the activation of PPARα by PFAS, expressed through benchmark dose (BMD), estimated with a benchmark ratio (BMR) of 1.5 relative to the solvent control. Computational descriptors were employed for modelling and feature selection was performed using a genetic algorithm. The model was validated through the use of statistical metrics representing goodness-of-fit in via cross validation, while y-scrambling was used to evaluate the existence of chance correlation.
2.1.	Date of QMRF	11 November 2025
2.2.	QMRF author(s) and contact details	Periklis Tsiros
2.3.	Date of QMRF update(s)	-NA-

2.4.	QMRF update(s)	-NA-
2.5.	Model developer(s) and contact details	Periklis Tsiros<br><br>ptsirostsib@gmail.com
2.6.	Date of model development and/or publication	2025
2.7.	Reference(s) to main scientific papers and/or software package	Alker et al.2025. Development of a QSAR model for predicting PPARα activation by PFAS based on human in vitro data of a comprehensive panel of legacy and novel PFAS, Archives of Toxicology, (Manuscript under review).
2.8.	Availability of information about the model	The detailed description of the model development and evaluation is provided in the corresponding publication:<br><br>Alker et al.2025. Development of a QSAR model for predicting PPARα activation by PFAS based on human in vitro data of a comprehensive panel of legacy and novel PFAS, Archives of Toxicology, (Manuscript under review).
2.9.	Availability of another QMRF for exactly the same model	No
**3**	**Defining the endpoint - OECD Principle 1: “A DEFINED ENDPOINT"**	**PRINCIPLE 1: “A DEFINED ENDPOINT". ENDPOINT refers to any physicochemical, biological, or environmental property/activity/effect that can be measured and therefore modelled. The intent of PRINCIPLE 1 (a (Q)SAR should be associated with a defined endpoint) is to ensure clarity in the endpoint being predicted by a given model, since a given endpoint could be determined by different experimental protocols and under different experimental conditions. It is therefore important to identify the experimental system and test conditions that is being modelled by the Q)SAR.**
3.1.	Species	Human Cells

3.2.	Endpoint	PPARα activation
3.3	Comment on endpoint	The endpoint refers to the activation of the human PPARα receptor following exposure to PFAS.
3.4.	Endpoint units	μM
3.5.	Dependent variable	PPARα activation expressed through a benchmark dose (BMD) using a benchmark ratio (BMR) of 1.5 relative to the solvent control.
3.6.	Experimental protocol	HEK293T cells were transiently transfected with a PPARα expression plasmid together with a PPAR-responsive luciferase reporter construct. Receptor activation was quantified using a dual-luciferase reporter (DLR) assay, and results were expressed as fold-change relative to control.
3.7.	Endpoint data quality and variability	The dataset details experimental PPARα induction data in response to at least 5 different concentrations of a panel of 34 PFAS congeners. Each data point summarizes the average of three biological replicates.
**4**	**Defining the algorithm - OECD Principle 2 : “AN UNAMBIGUOUS ALGORITHM”**	**PRINCIPLE 2: “AN UNAMBIGUOUS ALGORITHM”. The (Q)SAR estimate of an endpoint is the result of applying an ALGORITHM to a set of structural parameters which describe the chemical structure. The intent of PRINCIPLE 2 (a (Q)SAR should be associated with an unambiguous algorithm) is to ensure transparency in the model algorithm that generates predictions of an endpoint from information on chemical structure and/or physicochemical properties. In this context, algorithm refers to any mathematical equation, decision rule or output approach.**
4.1.	Type of model	Machine Learning Model

4.2.	Explicit algorithm	Linear regressor
4.3.	Descriptors in the model	Total of 7 descriptors:<br><br>AATS5dv, ATCS5dv, ATSC6c, GATS5p and two descriptors from extended connectivity fingerprints (ECFPs)
4.4.	Descriptor selection	Variance threshold was applied to remove features with zero variance. Then, for features with pearson correlation coefficient > 0.9, the first feature of the set was dropped.
4.5.	Algorithm and descriptor generation	Descriptors were generated using python modules
4.6.	Software name and version for descriptor generation	Rdkit v.2023.9.6 and Mordred v.2.0.5, accessed through Python v.3.10
4.7.	Chemicals/Descriptors ratio	34/7 = 4.9
**5**	**Defining the applicability domain - OECD Principle 3: “A DEFINED DOMAIN OF APPLICABILITY”**	**PRINCIPLE 3: “A DEFINED DOMAIN OF APPLICABILITY”. APPLICABILITY DOMAIN refers to the response and chemical structure space in which the model makes predictions with a given reliability. Ideally the applicability domain should express the structural, physicochemical and response space of the model. The CHEMICAL STRUCTURE (x variable) space can be expressed by information on physicochemical properties and/or structural fragments. The RESPONSE (y variable) can be any physicochemical, biological or environmental effect that is being predicted. According to PRINCIPLE 3 a (Q)SAR should be associated with a defined domain of applicability. Section 5 can be repeated (e.g., 5.a, 5.b, 5.c, etc) as many times as necessary if more than one method has been used to assess the applicability domain.**
5.1.	Description of the applicability domain of the model	The applicability domain (AD) was defined using a composite domain consisting of the Leverage method and he bounding box method.

5.2.	Method used to assess the applicability domain	For a substance to be within the composite applicability domain, it needs to be within the domain of both the leverage and bounding box methods. The applicability domain of the leverage method was assessed using the leverage (h) versus standardized residuals. The threshold for structural similarity was defined as:<br><br>$h^* = 3*(p+1)/n$<br><br>where p is the number of descriptors and n is the number of training compounds. Compounds with $h>h^*$ are considered outside the model's structural domain.<br><br>For bounding box, a compound is considered outside of the applicability domain if for any descriptor, the value is higher than the highest value or lower than the lowest value observed in the training dataset for the particular descriptor
5.3.	Software name and version for applicability domain assessment	japqotpy 7.0.
5.4.	Limits of applicability	The model is applicable to compounds that fall within the descriptor space defined by the training set. Predictions for structurally unrelated chemicals, compounds outside the calculated leverage threshold, or chemicals with physicochemical properties (e.g., molecular weight, chain length, functional groups) that fall outside the range observed in the training data may be associated with decreased reliability. In addition, predictions should only be interpreted within the biological context of PPARα activation in transfected HEK293T cells and may not directly translate to in vivo potency or systemic toxicity.
**6**	**Defining goodness-of-fit and robustness (internal validation) - OECD Principle 4: "APPROPRIATE MEASURES OF GOODNESS-OF-FIT, ROBUSTENESS AND PREDICTIVITY"**	**PRINCIPLE 4: "APPROPRIATE MEASURES OF GOODNESS-OF-FIT, ROBUSTENESS AND PREDICTIVITY". PRINCIPLE 4 expresses the need to perform validation to establish the performance of the model. GOODNESS-OF-FIT and ROBUSTNESS refer to the internal model performance.**

6.1.	Availability of the training set	The training set is available through the following publication: Alker et al.2025. Development of a QSAR model for predicting PPARα activation by PFAS based on human in vitro data of a comprehensive panel of legacy and novel PFAS, Archives of Toxicology, (Manuscript under review).
6.2.	Available information for the training set	Available information for the training set:<br>Descriptors for the PFAS.<br>CAS RN: Yes<br>Chemical Name: Yes<br>Smiles: Yes
6.3.	Data for each descriptor variable for the training set	All
6.4.	Data for the dependent variable for the training set	All
6.5.	Other information about the training set	-NA-
6.6.	Pre-processing of data before modelling	All data were normalised using the mean and standard deviation of each feature observed in the training set.
6.7.	Statistics for goodness-of-fit	R^2 = 0.92<br>MAE = 9.88<br>RMSE = 13.72
6.8.	Robustness - Statistics obtained by leave-one-out cross-validation	-NA-
6.9.	Robustness - Statistics obtained by leave-many-out cross-validation	10-fold cross validation:<br>R^2cv = 0.84<br>MAEcv = 13.95<br>RMSEcv = 19.72
6.10.	Robustness - Statistics obtained by Y-scrambling	10 random shufflings of the target train variable.<br>R^2 = -0.67<br><br>MAE = 23.87<br>RMSE = 29.93

6.11.	Robustness - Statistics obtained by bootstrap	-NA-
6.12.	Robustness - Statistics obtained by other methods	-NA-
**7**	**Defining predictivity (external validation) - OECD Principle 4: “APPROPRIATE MEASURES OF GOODNESS-OF-FIT, ROBUSTENESS AND PREDICTIVITY”**	**PRINCIPLE 4: “APPROPRIATE MEASURES OF GOODNESS-OF-FIT, ROBUSTENESS AND PREDICTIVITY”. PRINCIPLE 4 expresses the need to perform validation to establish the performance of the model. PREDICTIVITY refers to the external model validation. Section 7 can be repeated (e.g., 7.a, 7.b, 7.c, etc) as many times as necessary if more validation studies need to be reported in the QMRF.**
7.1.	Availability of the external validation set	Due to the limited size of the dataset, a separate external test set was not used.
7.2.	Available information for the external validation set	-NA-
7.3.	Data for each descriptor variable for the external validation set	-NA-
7.4.	Data for the dependent variable for the external validation set	-NA-
7.5.	Other information about the external validation set	-NA-
7.6.	Experimental design of test set	-NA-
7.7.	Predictivity - Statistics obtained by external validation	-NA-

7.8.	Predictivity - Assessment of the external validation set	-NA-
7.9.	Comments on the external validation of the model	-NA-
**8**	**Providing a mechanistic interpretation - OECD Principle 5: “A MECHANISTIC INTERPRETATION, IF POSSIBLE”**	**PRINCIPLE 5: “A MECHANISTIC INTERPRETATION, IF POSSIBLE”. According to PRINCIPLE 5, a (Q)SAR should be associated with a mechanistic interpretation, if possible.**
8.1.	Mechanistic basis of the model	-NA-
8.2.	A priori or a posteriori mechanistic interpretation	-NA-
8.3.	Other information about the mechanistic interpretation	-NA-
**9**	**Miscellaneous information**	
9.1.	Comments	None
9.2.	Bibliography	
9.3	Supporting information	
